# Association of maternal weight with *FADS* and *ELOVL* genetic variants and fatty acid levels- The PREOBE follow-up

**DOI:** 10.1371/journal.pone.0179135

**Published:** 2017-06-09

**Authors:** Andrea de la Garza Puentes, Rosa Montes Goyanes, Aida Maribel Chisaguano Tonato, Francisco José Torres-Espínola, Miriam Arias García, Leonor de Almeida, María Bonilla Aguirre, Marcela Guerendiain, Ana Isabel Castellote Bargalló, Maite Segura Moreno, Luz García-Valdés, Cristina Campoy, M. Carmen Lopez-Sabater, PREOBE team

**Affiliations:** 1 Department of Nutrition, Food Sciences and Gastronomy, Faculty of Pharmacy and Food Sciences, University of Barcelona, Barcelona, Spain; 2 Food Research and Analysis Institute, University of Santiago de Compostela, Santiago de Compostela, Spain; 3 CIBER Physiopathology of Obesity and Nutrition CIBERobn, Institute of Health Carlos III, Madrid, Spain; 4 Nutrition, Public Health School, Faculty of Health Sciences, University of San Francisco de Quito, Quito, Ecuador; 5 Centre of Excellence for Paediatric Research EURISTIKOS, University of Granada, Granada, Spain; 6 Department of Paediatrics, University of Granada, Granada, Spain; 7 School of Medicine, University of Chimborazo, Riobamba, Ecuador; 8 CIBER Epidemiology and Public Health CIBEResp, Institute of Health Carlos III, Madrid, Spain; University of Missouri Columbia, UNITED STATES

## Abstract

Single nucleotide polymorphisms (SNPs) in the genes encoding the fatty acid desaturase (*FADS*) and elongase (*ELOVL*) enzymes affect long-chain polyunsaturated fatty acid (LC-PUFA) production. We aimed to determine if these SNPs are associated with body mass index (BMI) or affect fatty acids (FAs) in pregnant women. Participants (n = 180) from the PREOBE cohort were grouped according to pre-pregnancy BMI: normal-weight (BMI = 18.5–24.9, n = 88) and overweight/obese (BMI≥25, n = 92). Plasma samples were analyzed at 24 weeks of gestation to measure FA levels in the phospholipid fraction. Selected SNPs were genotyped (7 in *FADS1*, 5 in *FADS2*, 3 in *ELOVL2* and 2 in *ELOVL5*). Minor allele carriers of rs174545, rs174546, rs174548 and rs174553 (*FADS1*), and rs1535 and rs174583 (*FADS2*) were nominally associated with an increased risk of having a BMI≥25. Only for the normal-weight group, minor allele carriers of rs174537, rs174545, rs174546, and rs174553 (*FADS1*) were negatively associated with AA:DGLA index. Normal-weight women who were minor allele carriers of *FADS* SNPs had lower levels of AA, AA:DGLA and AA:LA indexes, and higher levels of DGLA, compared to major homozygotes. Among minor allele carriers of *FADS2* and *ELOVL2* SNPs, overweight/obese women showed higher DHA:EPA index than the normal-weight group; however, they did not present higher DHA concentrations than the normal-weight women. In conclusion, minor allele carriers of *FADS* SNPs have an increased risk of obesity. Maternal weight changes the effect of genotype on FA levels. Only in the normal-weight group, minor allele carriers of *FADS* SNPs displayed reduced enzymatic activity and FA levels. This suggests that women with a BMI≥25 are less affected by *FADS* genetic variants in this regard. In the presence of *FADS2* and *ELOVL2* SNPs, overweight/obese women showed higher n-3 LC-PUFA production indexes than women with normal weight, but this was not enough to obtain a higher n-3 LC-PUFA concentration.

## Introduction

During pregnancy, the mother is the sole source of key nutrients for the fetus, such as n-6 and n-3 long-chain (LC) polyunsaturated fatty acids (PUFAs) [1–3]. Studies have shown that arachidonic acid (AA; C20:4n−6) and particularly docosahexaenoic acid (DHA; C22:6n−3), play an important role in many physiological conditions [4], such as neural and visual development [1, 3, 5]. Hyperlipidemia develops in normal pregnancy; accordingly, it has been observed that levels of fatty acid (FA), such as DHA, increase in maternal plasma during pregnancy. Nevertheless, a higher activity of the enzymes encoded by the *FADS* and *ELOVL* genes could also be related to this hyperlipidemia [6]. Due to the beneficial effects of n-3 LC-PUFAs, it is recommended to increase their intake during pregnancy [4]. This is in contrast with the effect of excessive n-6 FA intake, which may lead to maternal obesity and obesity-related complications such as an increased risk of cardiovascular disease [7, 8]. Moreover, obese pregnancies lead to a higher risk of obesity in newborn babies, thereby increasing the likelihood of lifelong obesity and obesity-related complications [9, 10].

Apart from diet, LC-PUFAs can be obtained via endogenous synthesis from their essential n-6 and n-3 PUFA precursors: linoleic acid (LA) and α-linolenic acid (ALA), respectively [1] (S1 Fig). This process requires desaturation and elongation reactions, which are catalyzed by delta-5 and delta-6 fatty acid desaturases (encoded by *FADS*1 and *FADS*2 genes, respectively) and elongases (encoded by *ELOVL* gene (elongation of very long chain fatty acids)) [1, 11, 12]. The n-3 and n-6 series compete for these enzymes since they participate in both pathways. Single nucleotide polymorphisms (SNPs) in these genes may affect LC-PUFA production, and consequently alter FA levels [12]. For instance, minor allele carriers of the *FADS1*, *FADS2* and *ELOVL2* genes, have been linked to lower LC-PUFA production, and consequently they show increased concentrations of substrates and decreased levels of products in the LC-PUFA metabolic pathway [1, 12–15].

Certain SNPs in the *FADS* gene have been linked to diseases such as coronary artery disease [16] and type 2 diabetes [17]. Obesity is related to these conditions and is already known to affect lipid metabolism during pregnancy [18]; however, to the best of our knowledge, maternal obesity has not yet been directly linked to SNPs in the *FADS* and *ELOVL* genes.

Current studies analyzing the impact of *FADS* and *ELOVL* polymorphisms on FA levels are not properly comparable because their analyses include different tissue samples, FAs and SNPs. Given the impact of both obesity and SNPs on LC-PUFA production, the association between obesity and SNPs in *FADS* and *ELOVL* genes is of interest. These nutritional studies involving pregnant women are of great importance since the synthesis of LC-PUFA is induced in pregnancy and therefore the SNPs might have a different effect on pregnant women than on the general population. Thus, the aim of this study was to determine if *FADS* and *ELOVL* genetic variants are associated with body mass index (BMI) or affect PUFA levels in pregnant women.

## Materials and methods

### Study design and participants

The study complies with the Declaration of Helsinki. The protocol was approved by the medical ethics committees of the Clinical University Hospital San Cecilio and the Mother–Infant Hospital in the city of Granada, Spain. Written informed consent was obtained from all the participants at the beginning of the study.

**Fig 1:**
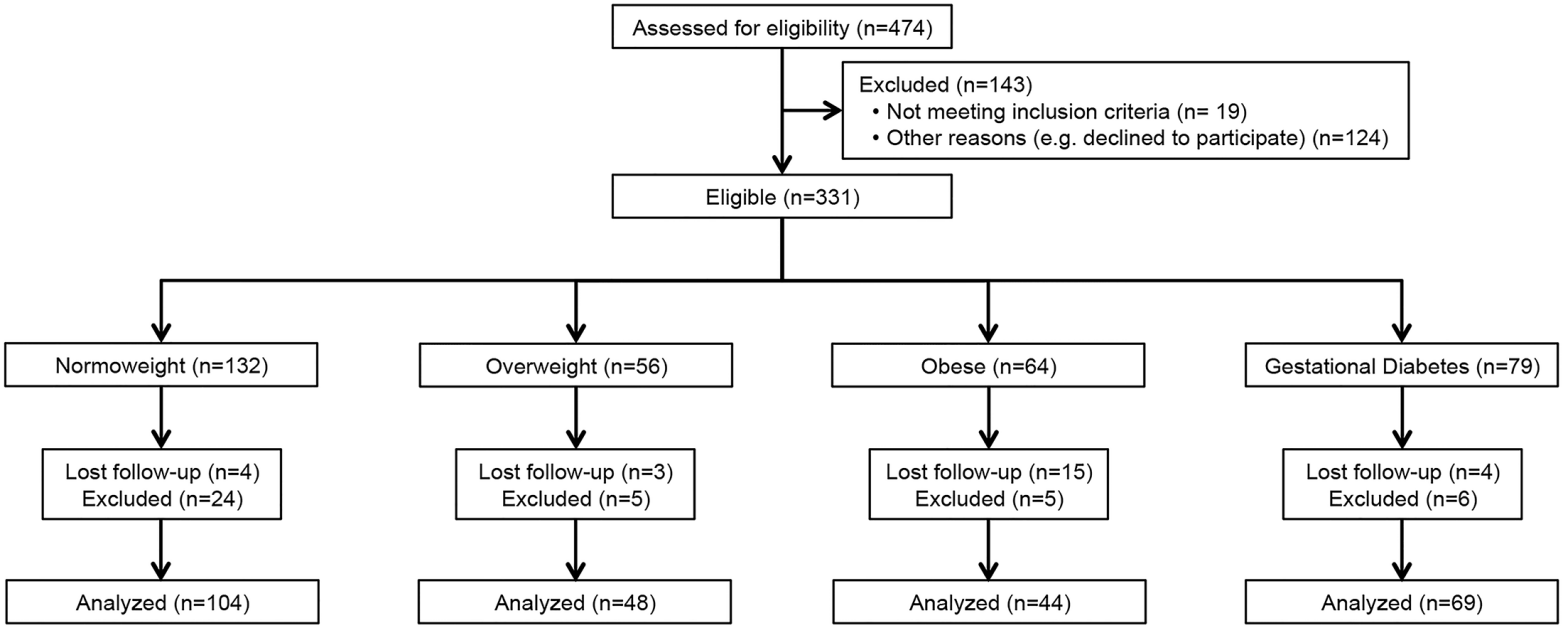
Participants in the PREOBE study and classification following BMI and gestational diabetes criteria.

Pregnant women (n = 180) were selected from the 331 individuals participating in the observational PREOBE cohort study (study of maternal nutrition and genetics on fetal adiposity programming) [19]. Participants were recruited at the Clinical University Hospital San Cecilio and the Mother–Infant Hospital in the city of Granada, Spain, where samples and information were also collected. Study design and information on PREOBE participants are exhibited in Fig 1.

The inclusion criteria were singleton pregnancy, gestation between 12 and 20 weeks at enrollment, and intention to deliver at the centers involved. Women were excluded if they were participating in other research studies, if they had been receiving drug treatment or supplements of either DHA or folate for more than the first 3 months of pregnancy, if they were suffering from disorders such as hypertension, pre-eclampsia, fetal intrauterine growth retardation, infections, hypo- or hyperthyroidism or hepatic renal diseases, or if they were following an extravagant or vegan diet. Using questionnaires and medical records, baseline and background characteristics were recorded such as maternal age, pre-pregnancy BMI, parity, smoking status, diet, alcohol drinking habits, socio-demographic information, educational level, and weight gain during pregnancy.

For this study, 180 pregnant women were divided into two groups according to their pre-pregnancy BMI, normal-weight (BMI = 18.5–24.9, n = 88) and overweight/obese (BMI≥25, n = 92). Plasma samples were collected at 24 weeks of gestation for FA analysis. Seventeen SNPs (7 in the *FADS1* gene, 5 in *FADS2*, 3 in *ELOVL2* and 2 in *ELOVL5*) out of the 32 initially selected were successfully genotyped and included in the analysis (S2 Table).

### DNA analysis

Maternal material for DNA analysis was collected by scraping the inside of the cheek of the pregnant participants with a buccal swab. Thirty-two SNPs in genes involved in lipid metabolism (*FADS1*, *FADS2*, *FADS3*, *ELOVL2*, *ELOVL5*, *ELOVL6* and *FASN*) were initially genotyped from 5 μl of maternal DNA mixed with 5 μl of 2X TaqMan^®^ OpenArray^®^ Genotyping Master Mix. The analysis was then performed with 3 μl of the mixture in a microplate using Taqman^®^ Open Array^®^ genotyping technology. The OpenArray^®^ instrument (which consists of a Dual Flat Block GeneAmp^®^ PCR System 9700, Bio-Rad^®^ thermal cycler with a Slide Chambers Dual-Block Alpha unit and Thermo Electron PX2 thermal cycler) and the corresponding OpenArray^®^ SNP Genotyping Analysis software, located at the *Autonomous University of Barcelona* (UAB), were used for the analysis. The genotyping required two phases: a thermal cycle (PCR amplification) and detection of the final fluorescence signal. The reagents used were supplied by Applied Biosystem (Foster City, CA, USA).

### Fatty acid analysis

At 24 weeks of gestation, blood was obtained by arm venipuncture. Plasma was separated by centrifugation and immediately frozen and stored at -80°C until analysis. FAs were determined in the phospholipid fraction using the method developed by Chisaguano et al. [20]. Plasma lipids were extracted using 2:1 dichloromethane:methanol and phospholipids were isolated using solid-phase extraction (SPE). FA methyl esters from plasma phospholipids were analyzed using fast gas chromatography with a flame ionization detector. The results were expressed as percentages of the total FAs detected.

We analyzed the FAs involved in enzymatic reactions encoded by the *FADS* and *ELOVL* genes (substrates, products or indexes (product/substrates)). Furthermore, we studied the n3:n6 ratios (eicosapentaenoic acid (EPA):AA and DHA:AA), associated with obesity risk [7, 21–23].

### Statistical analysis

Statistical analysis was performed using the SPSS software (version 20.0; SPSS Inc., Chicago, IL, USA). The Kolmogorov–Smirnov test was used to study the normal distribution of data; non-normal data were natural-log transformed. The agreement of genotype frequencies with Hardy-Weinberg equilibrium expectations was tested by the chi-square test. Due to the limited sample size, heterozygotes and homozygous for minor alleles, were analyzed as one group. SNPs were coded according to minor allele count (0 for major homozygotes and 1 for the carriers of at least one minor allele) and analyzed as a numerical variable. However, this codification implies an additive and dominant model. The associations between SNPs and FAs were analyzed using linear regression; while the associations between SNPs and obesity were analyzed by logistic regression. All associations were corrected for potential confounders such as age, education, smoking status and energy intake. FA levels were compared using univariate ANOVA. Since DHA supplementation was largely absent and it did not affect DHA levels in plasma, supplementation data were omitted for parsimony. The Bonferroni correction was applied to take multiple testing into account and p-value thresholds were set at 0.004, which was applied within each trait.

## Results

### Population characteristics

The characteristics of the groups are shown in Table 1. Normal-weight women were more likely to have a higher level of education and take DHA supplementation; while the overweight/obese group had a lower energy intake and weight gain during pregnancy. No differences were observed in dietary FA levels. S1 Table shows a comparison of plasma DHA concentrations at 24 weeks of gestation between pregnant women who took DHA supplementation and those who did not. After finding that the participants who reported taking DHA supplement (n = 17) did not have higher serum DHA concentrations (p = 0.636) than the participants who did not take supplements (n = 93), we decided to include all the subjects in the analysis regardless of supplement use.

**Table 1:** Characteristics of the population.

	NORMAL-WEIGHT (n = 88)	OVERWEIGHT/OBESE (n = 92)	P
	Mean (SD)	Mean (SD)	
**Age (years)**	30.91 (4.09)	30.64 (4.20)	0.665
**Pre-pregnancy BMI (kg/m2)**	22.00 (1.64)	30.33 (4.20)	<0.001*
**Weight Gain (kg)**	12.64 (5.47)	9.19 (6.29)	0.001*
**Education (%)**			
**<Highschool**	10.23%	15.38%	0.401
**Highschool**	15.91%	19.78%	
**>Highschool**	73.86%	64.84%	
**Smoking during pregnancy (%)**	21.92%	27.27%	0.447
**DHA supplementation during pregnancy (%)**	25.00%	7.61%	0.002*
**Dietary intakes**			
**Energy intake (kcal/d)**	2177.54 (335.41)	1938.07 (591.28)	**0.017**
**EPA intake (g/d)**	0.13 (0.13)	0.12 (0.10)	0.713
**DHA intake (g/d)**	0.25 (0.20)	0.27 (0.18)	0.646
**AA intakes (g/d)**	0.13 (0.07)	0.13 (0.06)	0.98

P-value derived from global ANOVA and significance level (p≤0.004) was adjusted for multiple testing by Bonferroni correction. P-values <0.05 are highlighted in bold and significant associations that persisted after Bonferroni correction are additionally denoted by stars (*p< 0.004). BMI: body mass index; AA: arachidonic acid; EPA: eicosapentaenoic acid; DHA: docosahexaenoic acid.

S2 Table presents the characteristics of the studied SNPs, including the distribution of participants in each allele group (major homozygotes, heterozygotes and minor homozygotes). Normal-weight women tended to be major homozygotes for all the *FADS1* and *FADS2* SNPs studied, while women in the overweight/obese group were mostly heterozygotes. Regarding *ELOVL2* SNPs, both groups presented mostly heterozygous alleles, and no pattern was observed for *ELOVL5* SNPs.

### Association between SNPs and fatty acids

Table 2 shows nominal and significant associations between PUFA levels and *FADS* and *ELOVL* SNPs after adjusting for age, education, smoking status and energy intake (the complete analysis can be found in S3 Table). The most significant associations (p≤0.004) were only found in the normal-weight group, where minor allele carriers of rs174537, rs174545, rs174546 and rs174553 (*FADS1*) were negatively associated with the AA:dihomo-gamma-linolenic acid (DGLA) index.

**Table 2:** Associations between plasma proportions of PUFAs and *FADS* and *ELOVL* polymorphisms.

Fatty acid	Gene	SNP *Major/minor allele*		NORMAL-WEIGHT			OVERWEIGHT/OBESE		
				N	β	P	N	β	P
***FADS1* indexes**									
**AA:DGLA**									
	*FADS1*	rs174537	*G/T*	22	-0.81	0.004*	18	-0.48	0.155
	*FADS1*	rs174545	*C/G*	28	-0.70	0.003*	17	-0.48	0.174
	*FADS1*	rs174546	*C/T*	28	-0.70	0.003*	19	-0.49	0.139
	*FADS1*	rs174553	*A/G*	28	-0.70	0.003*	19	-0.49	0.139
	*FADS1*	rs174547	*T/C*	23	-0.67	**0.013**	18	-0.48	0.155
***FADS2* indexes**									
**DGLA:LA**									
	*FADS2*	rs1535	*A/G*	26	0.56	**0.029**	18	0.42	0.226
	*FADS2*	rs174583	*C/T*	27	0.53	**0.040**	19	0.41	0.226
	*FADS2*	rs99780	*C/T*	25	0.54	**0.042**	18	0.42	0.226
***ELOVL2* indexes**									
**DHA:DPAn3**									
	*ELOVL2*	rs2236212	*G/C*	25	-0.05	0.806	19	-0.58	**0.016**
	*ELOVL2*	rs3798713	*G/C*	25	0.00	0.995	19	-0.58	**0.016**
**Fatty acids involved in *FADS1* indexes**									
**C20:3n6 (DGLA)**									
	*FADS1*	rs174537	*G/T*	22	0.75	**0.012**	18	0.53	0.119
	*FADS1*	rs174545	*C/G*	28	0.57	**0.028**	17	0.52	0.137
	*FADS1*	rs174546	*C/T*	28	0.57	**0.028**	19	0.52	0.118
	*FADS1*	rs174553	*A/G*	28	0.57	**0.028**	19	0.52	0.118
	*FADS1*	rs174547	*T/C*	23	0.60	**0.034**	18	0.53	0.119
**C20:4n6 (AA)**									
	*FADS1*	rs174545	*C/G*	28	-0.55	**0.042**	17	-0.01	0.977
	*FADS1*	rs174546	*C/T*	28	-0.55	**0.042**	19	-0.01	0.971
	*FADS1*	rs174553	*A/G*	28	-0.55	**0.042**	19	-0.01	0.971
**C18:3n3 (ALA)**									
	*FADS1*	rs174537	*G/T*	22	0.71	**0.027**	18	-0.09	0.790
	*FADS1*	rs174547	*T/C*	23	0.66	**0.026**	18	-0.09	0.790
**Fatty acids involved in *FADS2* indexes**									
**C18:3n6 (GLA)**									
	*FADS2*	rs174575	*C/G*	22	0.12	0.672	19	0.64	**0.031**
**C20:3n6 (DGLA)**									
	*FADS2*	rs1535	*A/G*	26	0.65	**0.010**	18	0.53	0.119
	*FADS2*	rs174583	*C/G*	27	0.62	**0.015**	19	0.52	0.118
**C18:3n3 (ALA)**									
	*FADS2*	rs174575	*C/G*	23	0.50	**0.043**	19	0.22	0.494
**C20:5n3 (EPA)**									
	*FADS2*	rs99780	*C/T*	25	0.52	**0.035**	17	0.11	0.749
**C22:5n3 (DPAn3)**									
	*FADS2*	rs99780	*C/T*	25	0.62	**0.011**	18	-0.41	0.173

Associations between SNPs and fatty acids were analyzed using linear regression. SNPs were coded according to minor allele count and analyzed as a numeric variable. "β" = beta per minor allele standardized per the major allele. All associations were adjusted for potential confounders such as age, education, smoking and energy intake. P-values <0.05 are highlighted in bold and significant associations that persisted after Bonferroni correction are additionally denoted by stars (*p≤0.004). LA: Linoleic Acid; GLA: γ-Linolenic Acid; DGLA: Dihomo-γ-Linolenic Acid; AA: Arachidonic Acid; ALA: α-linolenic Acid; EPA: Eicosapentaenoic acid; DPAn3: Docosapentaenoic acid n3; DHA: Docosahexaenoic acid.

### Association of *FADS* SNPs with obesity risk

After adjusting for age, education, smoking status and energy intake, participants who were minor allele carriers of rs174545, rs174546, rs174548 and rs174553 (*FADS1*), and rs1535 and rs174583 (*FADS2*) were nominally associated (p<0.05) with an increased risk of having a BMI≥25, compared with two major allele carriers (Table 3).

**Table 3:** Associations between *FADS* and *ELOVL* genes and maternal obesity.

Gene	SNP *Major/Minor alleles*		N	BMI≥25		BMI≥25c	
				OR	P	ORc	Pc
***FADS1***							
	rs174537	*G/T*	66	2.12	0.147	2.89	0.069
	rs174545	*C/G*	79	2.34	0.074	3.11	**0.032**
	rs174546	*C/T*	81	2.55	**0.047**	3.28	**0.025**
	rs174548	*C/G*	82	3.11	**0.015**	3.18	**0.021**
	rs174553	*A/G*	82	2.64	**0.039**	3.44	**0.019**
	rs174561	*T/C*	44	0.56	0.351	0.43	0.217
	rs174547	*T/C*	66	1.96	0.191	2.75	0.082
***FADS2***							
	rs1535	*A/G*	77	2.58	**0.048**	3.42	**0.025**
	rs174575	*C/G*	70	1.57	0.351	1.77	0.273
	rs174583	*C/T*	79	2.62	**0.046**	3.38	**0.024**
	rs99780	*C/T*	71	1.75	0.261	2.37	0.122
	rs174602	*T/C*	46	1.55	0.475	1.18	0.812
***ELOVL2***							
	rs2236212	*G/C*	76	0.76	0.568	0.88	0.805
	rs3798713	*G/C*	75	0.67	0.430	0.81	0.708
	rs953413	*A/G*	58	1.00	1.000	0.58	0.425
***ELOVL5***							
	rs2397142	*C/G*	79	0.78	0.573	0.75	0.556
	rs9395855	*T/G*	54	0.50	0.316	0.50	0.345

The association between SNPs and obesity risk was analyzed with logistic regression. SNPs were coded according to minor allele count and analyzed as numeric variable. BMI = Body Mass Index. OR = odds ratio per minor allele with the major allele as reference. ORc and Pc = are corrected values after adjustment for age, education, smoking and energy intake. Nominal associations are highlighted in bold (p<0.05).

### Fatty acid levels according to genotype and weight

Plasma LC-PUFA levels according to genotypes are presented in Tables 4 and 5 (the complete analysis can be found in S4 Table). Significant differences were found for normal-weight women: minor allele carriers of *FADS1* SNPs showed lower AA level and both AA:DGLA and AA:LA indexes than major homozygotes; while minor allele carriers of *FADS2* SNPs showed lower AA and higher DGLA levels (p≤0.004). Overweight/obese women presented the same tendencies, but without the results reaching statistical significance. No differences were found between genotypes of *ELOVL* SNPs.

**Table 4:** *FADS1*, *FADS2 and ELOVL2* enzymatic indexes according to maternal SNPs and LC-PUFA levels in plasma.

*Gene*	SNP *Major/minor allele*		NORMAL-WEIGHT					OVERWEIGHT/OBESITY					P (*MM*)	P (*Mm+mm*)
			*MM*		*Mm+mm*		P	*MM*		*Mm+mm*		P		
			N	Mean (SD)	N	Mean (SD)		N	Mean (SD)	N	Mean (SD)			
***FADS1* indexes**														
**AA:LA**														
*FADS1*	rs174537	*G/T*	15	0.45 (0.08)	23	0.39 (0.09)	**0.038**	12	0.46 (0.07)	21	0.43 (0.08)	0.390	0.811	0.085
*FADS1*	rs174545	*C/G*	22	0.46 (0.09)	27	0.38 (0.08)	0.003*	13	0.47 (0.07)	22	0.42 (0.08)	0.071	0.877	0.171
*FADS1*	rs174546	*C/T*	22	0.46 (0.09)	27	0.38 (0.08)	0.003*	13	0.47 (0.07)	25	0.42 (0.08)	0.098	0.877	0.109
*FADS1*	rs174548	*C/G*	24	0.45 (0.08)	25	0.39 (0.09)	**0.031**	13	0.45 (0.08)	25	0.43 (0.08)	0.553	0.969	0.115
*FADS1*	rs174553	*A/G*	22	0.46 (0.09)	27	0.38 (0.08)	0.003*	13	0.47 (0.07)	25	0.42 (0.08)	0.098	0.877	0.109
*FADS1*	rs174547	*T/C*	15	0.45 (0.08)	23	0.39 (0.09)	**0.040**	12	0.46 (0.07)	20	0.43 (0.08)	0.427	0.950	0.124
**AA:DGLA**														
*FADS1*	rs174537	*G/T*	15	3.14 (0.70)	23	2.20 (0.60)	<0.001*	12	3.57 (1.73)	21	2.55 (0.80)	**0.032**	0.414	0.107
*FADS1*	rs174545	*C/G*	22	3.12 (0.65)	27	2.25 (0.57)	<0.001*	13	3.48 (1.67)	22	2.51 (0.78)	**0.025**	0.362	0.183
*FADS1*	rs174546	*C/T*	22	3.12 (0.65)	27	2.25 (0.57)	<0.001*	13	3.48 (1.67)	25	2.54 (0.74)	**0.021**	0.362	0.120
*FADS1*	rs174548	*C/G*	24	2.97 (0.65)	25	2.33 (0.70)	0.002*	13	3.42 (1.69)	25	2.57 (0.75)	**0.037**	0.244	0.246
*FADS1*	rs174553	*A/G*	22	3.12 (0.65)	27	2.25 (0.57)	<0.001*	13	3.48 (1.67)	25	2.54 (0.74)	**0.021**	0.362	0.120
*FADS1*	rs174547	*T/C*	15	3.20 (0.67)	23	2.27 (0.55)	<0.001*	12	3.54 (1.73)	20	2.56 (0.82)	**0.037**	0.291	0.194
***FADS2* indexes**														
**DGLA:LA**														
*FADS2*	rs1535	*A/G*	20	0.15 (0.04)	25	0.18 (0.05)	**0.011**	13	0.15 (0.04)	23	0.18 (0.05)	0.115	0.877	0.798
*FADS2*	rs174575	*C/G*	24	0.15 (0.04)	16	0.19 (0.06)	**0.017**	21	0.17 (0.06)	14	0.17 (0.05)	0.943	0.276	0.315
*FADS2*	rs174583	*C/T*	19	0.15 (0.04)	27	0.18 (0.05)	**0.036**	13	0.15 (0.04)	25	0.18 (0.05)	0.130	0.967	0.907
*FADS2*	rs99780	*C/T*	15	0.15 (0.04)	26	0.18 (0.05)	**0.037**	12	0.15 (0.05)	22	0.18 (0.05)	0.076	0.953	0.961
**AA:LA**														
*FADS2*	rs1535	*A/G*	20	0.45 (0.08)	25	0.39 (0.09)	**0.025**	13	0.47 (0.07)	23	0.42 (0.08)	0.128	0.504	0.170
*FADS2*	rs174583	*C/T*	19	0.44 (0.08)	27	0.38 (0.08)	**0.019**	13	0.47 (0.07)	25	0.42 (0.08)	0.098	0.447	0.109
**DHA:EPA**														
*FADS2*	rs1535	*A/G*	20	15.02 (5.64)	25	14.66 (5.92)	0.840	13	19.69 (6.63)	23	20.89 (7.87)	0.655	**0.045**	**0.004†**
*FADS2*	rs174575	*C/G*	24	16.41 (5.99)	16	13.41 (5.21)	0.119	21	20.99 (6.36)	14	20.27 (8.32)	0.778	**0.018**	**0.012**
*FADS2*	rs174583	*C/T*	19	15.02 (5.64)	27	14.48 (5.71)	0.753	13	19.69 (6.63)	25	20.58 (7.89)	0.737	**0.045**	**0.003†**
*FADS2*	rs99780	*C/T*	15	16.22 (5.52)	26	13.71 (4.76)	0.138	12	20.50 (6.29)	22	20.19 (8.14)	0.911	0.078	**0.002†**
*FADS2*	rs174602	*T/C*	19	16.06 (5.17)	13	14.76 (6.12)	0.531	23	19.97 (6.82)	14	18.02 (6.37)	0.393	**0.046**	0.199
**DHA:DPAn3**														
*FADS2*	rs1535	*A/G*	20	10.84 (1.96)	25	10.03 (2.40)	0.232	13	11.14 (2.26)	23	11.58 (2.26)	0.577	0.687	**0.027**
*FADS2*	rs174575	*C/G*	24	10.58 (2.21)	16	10.15 (2.39)	0.574	21	11.23 (2.29)	14	12.20 (2.28)	0.230	0.334	**0.026**
*FADS2*	rs174583	*C/T*	19	10.76 (1.98)	27	10.13 (2.33)	0.350	13	11.14 (2.26)	25	11.53 (2.50)	0.642	0.613	**0.044**
*FADS2*	rs99780	*C/T*	15	11.04 (2.07)	26	9.83 (2.17)	0.090	12	11.42 (2.11)	22	11.41 (2.45)	0.990	0.640	**0.024**
***ELOVL2* indexes**														
**DPAn3:EPA**														
*ELOVL2*	rs2236212	*G/C*	14	1.51 (0.51)	29	1.48 (0.67)	0.876	13	1.66 (0.71)	22	1.88 (0.71)	0.388	0.530	**0.047**
*ELOVL2*	rs3798713	*G/C*	11	1.48 (0.52)	31	1.44 (0.67)	0.863	12	1.67 (0.74)	25	1.88 (0.70)	0.410	0.476	**0.021**
**DHA:EPA**														
*ELOVL2*	rs2236212	*G/C*	14	16.39 (6.29)	28	14.45 (5.43)	0.307	13	20.58 (8.33)	22	20.05 (7.02)	0.841	0.151	**0.003†**
*ELOVL2*	rs3798713	*G/C*	11	15.48 (5.10)	30	13.96 (5.33)	0.418	12	20.76 (8.67)	24	20.13 (7.07)	0.819	0.094	**<0.001†**
*ELOVL2*	rs953413	*A/G*	10	15.33 (5.93)	25	14.78 (5.23)	0.789	7	21.14 (7.60)	20	20.88 (7.57)	0.939	0.096	**0.003†**
**DHA:DPAn3**														
*ELOVL2*	rs2236212	*G/C*	14	10.87 (2.20)	29	10.14 (2.32)	0.337	13	12.57 (1.53)	22	10.77 (2.67)	**0.032**	**0.029**	0.375
*ELOVL2*	rs3798713	*G/C*	11	10.68 (2.24)	31	10.10 (2.20)	0.460	12	12.64 (1.58)	25	10.78 (2.55)	**0.028**	**0.024**	0.291
*ELOVL2*	rs953413	*A/G*	10	9.83 (2.22)	26	10.38 (1.99)	0.478	7	10.68 (2.80)	20	12.04 (2.09)	0.183	0.493	**0.008**

P-value derived from global ANOVA and significance level (p≤0.004) was adjusted for multiple testing by Bonferroni correction. Data are means of FAs expressed as percentages of the total phospholipid profile (standard error). P-values <0.05 are highlighted in bold and significant associations that persisted after Bonferroni correction are additionally denoted by stars or daggers (p≤0.004). *Indicates significant differences within each group of weight and † Indicates significant differences between groups of weight. Major allele: M; minor allele: m; LA: Linoleic Acid; GLA: γ-Linolenic Acid; DGLA: Dihomo-γ-Linolenic Acid; AA: Arachidonic Acid; AdA: Adrenic Acid; DPAn6: Docosapentaenoic acid n6; ALA: α-linolenic Acid; EPA: Eicosapentaenoic acid; DPAn3: Docosapentaenoic acid n3; DHA: Docosahexaenoic acid.

**Table 5:** Substrates and products of enzymatic indexes according to maternal SNPs and LC-PUFA levels in plasma.

*Gene*	SNP *Major/minor allele*		NORMAL-WEIGHT					OVERWEIGHT/OBESITY					P (*MM*)	P (*Mm+mm*)
			*MM*		*Mm+mm*		P	*MM*		*Mm+mm*		P		
			N	Mean (SD)	N	Mean (SD)		N	Mean (SD)	N	Mean (SD)			
**Fatty acids involved in *FADS1* indexes**														
**C20:3n-6 (DGLA)**														
*FADS1*	rs174537	*G/T*	15	3.51 (0.81)	23	4.27 (0.85)	**0.010**	12	3.31 (0.80)	21	4.18 (0.96)	**0.012**	0.529	0.754
*FADS1*	rs174545	*C/G*	22	3.50 (0.79)	27	4.14 (0.86)	**0.009**	13	3.39 (0.81)	22	4.15 (0.94)	**0.021**	0.691	0.976
*FADS1*	rs174546	*C/T*	22	3.50 (0.79)	27	4.14 (0.86)	**0.009**	13	3.39 (0.81)	25	4.11 (0.90)	**0.021**	0.691	0.893
*FADS1*	rs174548	*C/G*	24	3.59 (0.79)	25	4.10 (0.93)	**0.041**	13	3.38 (0.81)	25	4.11 (0.90)	**0.019**	0.431	0.969
*FADS1*	rs174553	*A/G*	22	3.50 (0.79)	27	4.14 (0.86)	**0.009**	13	3.39 (0.81)	25	4.11 (0.90)	**0.021**	0.691	0.893
*FADS1*	rs174547	*T/C*	15	3.61 (0.78)	23	4.17 (0.76)	**0.036**	12	3.31 (0.80)	20	4.17 (0.98)	**0.016**	0.335	0.980
**C20:4n-6 (AA)**														
*FADS1*	rs174537	*G/T*	15	10.55 (1.33)	23	9.02 (1.54)	0.003*	12	10.53 (0.89)	21	10.04 (1.36)	0.273	0.973	**0.026**
*FADS1*	rs174545	*C/G*	22	10.50 (1.39)	27	8.96 (1.49)	0.001*	13	10.64 (0.93)	22	9.83 (1.31)	0.061	0.759	**0.037**
*FADS1*	rs174546	*C/T*	22	10.50 (1.39)	27	8.96 (1.49)	0.001*	13	10.64 (0.93)	25	9.90 (1.31)	0.080	0.759	**0.019**
*FADS1*	rs174548	*C/G*	24	10.28 (1.39)	25	9.05 (1.64)	**0.007**	13	10.40 (1.02)	25	10.03 (1.33)	0.389	0.790	**0.025**
*FADS1*	rs174553	*A/G*	22	10.50 (1.39)	27	8.96 (1.49)	0.001*	13	10.64 (0.93)	25	9.90 (1.31)	0.080	0.759	**0.019**
*FADS1*	rs174561	*T/C*	15	9.88 (1.32)	14	9.30 (1.12)	0.212	20	10.27 (1.48)	17	10.33 (1.54)	0.910	0.423	**0.046**
*FADS1*	rs174547	*T/C*	15	10.49 (1.33)	23	9.18 (1.47)	**0.009**	12	10.53 (0.89)	20	10.00 (1.38)	0.243	0.917	0.067
**C18:3n-3 (ALA)**														
*FADS1*	rs174537	*G/T*	15	0.12 (0.04)	23	0.16 (0.04)	**0.021**	12	0.11 (0.03)	21	0.11 (0.04)	0.632	0.496	**<0.001†**
*FADS1*	rs174545	*C/G*	22	0.12 (0.04)	27	0.15 (0.04)	**0.018**	13	0.12 (0.03)	22	0.11 (0.04)	0.532	0.536	**0.001†**
*FADS1*	rs174546	*C/T*	22	0.12 (0.04)	27	0.15 (0.04)	**0.018**	13	0.12 (0.03)	25	0.11 (0.04)	0.580	0.536	**0.001†**
*FADS1*	rs174548	*C/G*	24	0.13 (0.04)	22	0.15 (0.04)	0.146	12	0.12 (0.03)	25	0.11 (0.04)	0.454	0.371	**0.001**
*FADS1*	rs174553	*A/G*	22	0.12 (0.04)	27	0.15 (0.04)	**0.018**	13	0.12 (0.03)	25	0.11 (0.04)	0.580	0.536	**0.001†**
*FADS1*	rs174561	*T/C*	15	0.13 (0.04)	14	0.13 (0.03)	0.695	20	0.12 (0.05)	17	0.11 (0.03)	0.497	0.397	**0.024**
*FADS1*	rs174547	*T/C*	15	0.12 (0.04)	23	0.15 (0.04)	**0.027**	12	0.11 (0.03)	20	0.11 (0.04)	0.734	0.444	**<0.001†**
**C20:5n3 (EPA)**														
*FADS1*	rs174561	*T/C*	15	0.33 (0.14)	13	0.31 (0.11)	0.683	20	0.26 (0.11)	17	0.23 (0.10)	0.491	0.092	**0.044**
**Fatty acids involved in *FADS2* indexes**														
**C20:3n-6 (DGLA)**														
*FADS2*	rs1535	*A/G*	20	3.41 (0.77)	25	4.23 (0.82)	0.001*	13	3.39 (0.81)	23	4.15 (0.92)	**0.019**	0.927	0.736
*FADS2*	rs174575	*C/G*	24	3.57 (0.71)	16	4.28 (0.97)	**0.012**	21	3.82 (1.30)	14	3.90 (0.90)	0.805	0.358	0.283
*FADS2*	rs174583	*C/T*	19	3.47 (0.75)	27	4.14 (0.86)	**0.009**	13	3.39 (0.81)	25	4.11 (0.90)	**0.021**	0.762	0.893
*FADS2*	rs99780	*C/T*	15	3.51 (0.78)	26	4.16 (0.89)	**0.025**	12	3.35 (0.84)	22	4.14 (0.94)	**0.022**	0.613	0.941
*FADS2*	rs174602	*T/C*	19	3.77 (0.82)	13	3.50 (0.85)	0.387	23	3.95 (0.76)	14	4.19 (0.54)	0.297	0.463	**0.018**
**C20:4n-6 (AA)**														
*FADS2*	rs1535	*A/G*	20	10.31 (1.30)	25	9.07 (1.50)	**0.005**	13	10.64 (0.93)	23	9.92 (1.35)	0.102	0.439	**0.044**
*FADS2*	rs174583	*C/T*	19	10.33 (1.34)	27	8.96 (1.49)	0.003*	13	10.64 (0.93)	25	9.90 (1.31)	0.080	0.482	**0.019**
*FADS2*	rs99780	*C/T*	15	10.39 (1.48)	26	9.15 (1.51)	**0.015**	12	10.54 (0.91)	22	10.02 (1.40)	0.254	0.757	**0.046**
**C18:3n-3 (ALA)**														
*FADS2*	rs1535	*A/G*	20	0.13 (0.04)	25	0.15 (0.04)	**0.025**	13	0.12 (0.03)	23	0.11 (0.04)	0.448	0.500	**<0.001†**
*FADS2*	rs174575	*C/G*	24	0.13 (0.04)	16	0.16 (0.04)	**0.013**	21	0.11 (0.03)	14	0.11 (0.03)	0.972	**0.035**	**<0.001†**
*FADS2*	rs174583	*C/T*	19	0.13 (0.04)	27	0.15 (0.04)	**0.033**	13	0.12 (0.03)	25	0.11 (0.04)	0.580	0.498	**0.001†**
*FADS2*	rs99780	*C/T*	15	0.13 (0.04)	26	0.15 (0.04)	0.060	12	0.12 (0.03)	22	0.11 (0.04)	0.584	0.576	**0.001†**
**C20:5n-3 (EPA)**														
*FADS2*	rs174602	*T/C*	19	0.31 (0.14)	12	0.32 (0.10)	0.780	23	0.23 (0.09)	14	0.27 (0.13)	0.347	**0.040**	0.241
**C22:5n-3 (DPAn3)**														
*FADS2*	rs1535	*A/G*	20	0.41 (0.10)	25	0.42 (0.06)	0.730	13	0.42 (0.11)	23	0.37 (0.10)	0.190	0.887	**0.034**
*FADS2*	rs174575	*C/G*	24	0.40 (0.09)	16	0.44 (0.05)	0.056	21	0.39 (0.11)	14	0.37 (0.10)	0.632	0.872	**0.033**
*FADS2*	rs174583	*C/T*	19	0.40 (0.10)	27	0.42 (0.06)	0.388	13	0.42 (0.11)	25	0.37 (0.10)	0.158	0.674	**0.018**
*FADS2*	rs99780	*C/T*	15	0.38 (0.07)	26	0.44 (0.08)	**0.017**	12	0.42 (0.12)	22	0.39 (0.10)	0.429	0.297	**0.043**
**C22:6n-3 (DHA)**														
*FADS2*	rs1535	*A/G*	20	4.48 (1.35)	25	4.23 (1.04)	0.496	13	4.56 (0.98)	23	4.18 (0.93)	0.255	0.841	0.869
*FADS2*	rs174575	*C/G*	24	4.19 (1.22)	16	4.50 (1.02)	0.400	21	4.25 (0.92)	14	4.44 (1.01)	0.573	0.856	0.859
*FADS2*	rs174583	*C/T*	19	4.31 (1.16)	27	4.29 (1.06)	0.955	13	4.56 (0.98)	25	4.11 (0.93)	0.169	0.528	0.511
*FADS2*	rs99780	*C/T*	15	4.14 (0.95)	26	4.33 (1.19)	0.599	12	4.64 (0.98)	22	4.26 (0.88)	0.260	0.188	0.835
*FADS2*	rs174602	*T/C*	19	4.32 (1.09)	13	4.52 (1.22)	0.635	23	4.10 (0.91)	14	4.19 (1.02)	0.794	0.480	0.450
**Fatty acids involved in *ELOVL2* indexes**														
**C20:5n-3 (EPA)**														
*ELOVL2*	rs2236212	*G/C*	14	0.31 (0.12)	29	0.33 (0.15)	0.776	13	0.27 (0.23)	22	0.24 (0.11)	0.612	0.571	**0.032**
*ELOVL2*	rs3798713	*G/C*	11	0.32 (0.13)	31	0.34 (0.15)	0.765	12	0.28 (0.24)	25	0.24 (0.11)	0.490	0.626	**0.011**
**C22:5n-3 (DPAn3)**														
*ELOVL2*	rs2236212	*G/C*	14	0.43 (0.10)	29	0.41 (0.07)	0.424	13	0.34 (0.06)	22	0.41 (0.12)	**0.047**	**0.011**	0.787
*ELOVL2*	rs3798713	*G/C*	11	0.43 (0.11)	31	0.41 (0.07)	0.465	12	0.35 (0.06)	25	0.41 (0.12)	0.099	**0.033**	0.992
**C22:6n-3 (DHA)**														
*ELOVL2*	rs2236212	*G/C*	14	4.64 (1.32)	29	4.10 (0.98)	0.139	13	4.30 (0.99)	22	4.28 (0.97)	0.942	0.466	0.514
*ELOVL2*	rs3798713	*G/C*	11	4.60 (1.49)	31	4.10 (0.91)	0.200	12	4.41 (0.94)	25	4.24 (0.96)	0.617	0.728	0.565
*ELOVL2*	rs953413	*A/G*	10	3.82 (0.83)	26	4.31 (1.22)	0.250	7	3.85 (0.73)	20	4.61 (0.92)	0.059	0.929	0.357
**Fatty acids involved in *ELOVL5* indexes**														
**C18:3n-3 (ALA)**														
*ELOVL5*	rs2397142	*C/G*	23	0.13 (0.04)	20	0.15 (0.04)	0.168	19	0.11 (0.03)	16	0.11 (0.04)	0.605	**0.022**	**0.016**
*ELOVL5*	rs9395855	*T/G*	6	0.16 (0.05)	25	0.14 (0.04)	0.405	8	0.10 (0.02)	20	0.11 (0.04)	0.519	**0.010**	**0.047**
**C20:5n-3 (EPA)**														
*ELOVL5*	rs2397142	*C/G*	24	0.33 (0.14)	22	0.34 (0.14)	0.855	18	0.21 (0.09)	17	0.29 (0.22)	0.168	**0.004†**	0.411

P-value derived from global ANOVA and significance level (p≤0.004) was adjusted for multiple testing by Bonferroni correction. Data are means of FAs expressed as percentages of the total phospholipid profile (standard error). P-values <0.05 are highlighted in bold and significant associations that persisted after Bonferroni correction are additionally denoted by stars or daggers (p≤0.004). *Indicates significant differences within each group of weight and † Indicates significant differences between groups of weight. Major allele: M; minor allele: m; LA: Linoleic Acid; GLA: γ-Linolenic Acid; DGLA: Dihomo-γ-Linolenic Acid; AA: Arachidonic Acid; AdA: Adrenic Acid; DPAn6: Docosapentaenoic acid n6; ALA: α-linolenic Acid; EPA: Eicosapentaenoic acid; DPAn3: Docosapentaenoic acid n3; DHA: Docosahexaenoic acid.

Significant differences were also shown when comparing groups of weight (p≤0.004). Among the minor allele carriers of rs1535, rs174583 and rs99780 (*FADS2*), and rs2236212, rs3798713 and rs953413 (*ELOVL2*), overweight/obese women showed a higher DHA:EPA index than those in the normal-weight group (Table 4). Meanwhile, among minor alleles carriers of rs174537, rs174545, rs174546, rs174553 and rs174547 (*FADS1*), and rs1535, rs174575, rs174583 and rs99780 (*FADS2*), normal-weight women had higher levels of the substrate ALA than those in the overweight/obese group. In addition, among the major homozygotes of rs2397142 (*ELOVL5*), normal-weight women presented higher levels of EPA than women who were overweight/obese (Table 5).

Maternal plasma n3:n6 ratios, according to genotype, are presented in S5 Table. Among the minor allele carriers of rs3798713 (*ELOVL2*), normal-weight women had significantly higher EPA:AA ratio than those who were overweight/obese. This trend was also found in all gene clusters studied, even when comparing major homozygotes.

## Discussion

The present study analyzed the effect of *FADS* and *ELOVL* genetic variants on a broad FA profile. To the best of our knowledge, this is the first study to explore associations between *FADS* and *ELOVL* SNPs, FA levels and maternal pre-pregnancy weight. Despite a lack of studies analyzing the association between obesity risk and *FADS* or *ELOVL* polymorphisms, some authors have observed that minor alleles of rs174547 (*FADS1*) confer a higher risk of obesity-related conditions, such as increased triglyceride levels and decreased high-density lipoprotein cholesterol concentrations [24–26], as well as an increased risk of coronary disease [26, 27]. In the present study, we found that women who carried at least one minor allele of the *FADS1* and *FADS2* SNPs, were associated with a higher risk of having a BMI≥25 than homozygotes for the major allele. This association could explain why most of the overweight/obese women carried one minor allele copy and normal-weight women were mostly homozygous for the major alleles. Both weight groups showed very similar distributions within the allele groups of *ELOVL* SNPs; thus, we did not expect to find any associations between *ELOVL* genotypes and weight.

In line with other studies [5, 12], we found that the *FADS1* and *FADS2* SNPs were associated with FAs, mainly from the n-6 series, and less with those from the n-3 series. The only significant association (p-value ≤0.004) was found in the normal-weight group, where minor alleles of *FADS1* were associated with a lower AA:DGLA index. Several nominal associations were also found; nevertheless, they were mainly in normal-weight women and the n-6 series. Regarding the overweight/obese group, minor allele carriers of rs2236212 and rs3798713 (*ELOVL2)* were nominally associated with a lower DHA:n-3 docosapentaenoic acid (DPAn3) index. Barman et al. observed results similar to this last association, but their significance did not persist after correction either [12].

We also observed that *FADS* genetic variants affected FA concentrations. Normal-weight women who were minor allele carriers of *FADS1* SNPs had significantly lower levels of product (AA) and indexes (AA:LA and AA:DGLA) than major homozygotes. Consistently with this, nominal differences were also observed; minor allele carriers of *FADS1* SNPs showed nominally lower substrates (DGLA and ALA). This was previously reported by other authors [1, 5, 12, 13, 28, 29], who observed that minor allele carriers in *FADS* displayed lower FA indexes and products, and increased amounts of substrates. Overweight/obese women with *FADS1* SNPs showed the same trends and some nominal differences in DGLA and AA:DGLA, but none of them with a p-value ≤0.004. Other studies [1, 28, 29] have also found that *FADS2* SNPs were related to lower levels of AA and lower AA:LA index. This supports our findings for the normal-weight group, where minor allele carriers of *FADS2* SNPs had significantly lower levels of AA and higher DGLA. We also found nominal differences consistent with the previous results (lower AA:LA index and higher ALA and DPAn3). Similarly to other studies [30], the DGLA:LA index was nominally higher in minor allele carriers. This could be because the DGLA:LA index precedes the AA:LA index and, therefore, a lower AA:LA index would cause an accumulation of DGLA, thereby “increasing” the DGLA:LA index. Overweight/obese women who were minor allele carriers of *FADS2* SNPs only showed nominally higher levels of DGLA than major homozygotes.

As shown previously, the *FADS1* and *FADS2* genetic variants were found to affect mainly the n-6 FAs, even though desaturases and elongases work on both the n-6 and n-3 series. Several studies have observed that the DHA status, or that of the n-3 series, is less influenced by genetic variants in the *FADS* genes [1, 5, 12, 13, 28, 31]. One possible explanation for this is that the final conversion step from DPA to DHA requires translocation to the peroxisomes (which is not performed in the endoplasmic reticulum where the other reactions occur) [1], making DHA the least efficiently synthesized n-3 LC-PUFA. Therefore, the influence of SNPs might not ultimately affect DHA levels [1, 32]. It has also been postulated that DHA supplementation during pregnancy could reduce dependence on endogenous DHA synthesis [1]. However, previous studies have reported that increased dietary intake of EPA and DHA is linked to higher *FADS1* and lower *FADS2* activities, suggesting that endogenous LC-PUFA production remains active and is possibly enhanced, despite additional dietary intake of LC-PUFAs [1, 33]. Moreover, in our study, we observed no differences in dietary intake of EPA, DHA or AA between the two BMI groups; and although normal-weight pregnant women were more likely to take DHA supplementation, it was very unusual and this supplementation did not affect DHA level in plasma.

Our results suggest that BMI modifies genotype responses. According to *FADS* SNPs, n-6 FAs showed the same effects in the 2 groups studied, but significant effects were only found in the group of normal-weight women. This suggests that overweight/obese women are less affected. This could explain why women in the normal-weight group had significantly higher n-3 substrate (ALA) levels and nominally lower n-6 product (AA) levels than overweight/obese women, only when we compared minor allele carriers of SNPs in *FADS1* and *FADS2*. Additionally, among the minor allele carriers of *FADS2*, overweight/obese women showed significantly higher DHA:EPA index than the normal-weight group (the DHA:DPAn3 index showed the same trend). This suggests that minor alleles of *FADS2* SNPS in overweight/obese women could have a positive impact on n-3 LC-PUFA production. Furthermore, among major homozygotes, overweight/obese women also showed nominally higher DHA:EPA index than those in the normal-weight group. Perhaps, regardless of genetic variants in the *FADS* gene, a high BMI could be linked to increased activity of enzymes involved in n-3 FA synthesis. Nevertheless, there were no differences in amounts of DHA between the weight groups, suggesting that even if enzymatic activity in n-3 FA production is increased in overweight/obese individuals, it is not enough to elicit greater n-3 LC-PUFA levels than in normal-weight subjects.

We analyzed 3 *ELOVL2* SNPs (rs2236212, rs3798713 and rs953413) and found that their genetic variants only affected n-3 FA levels in overweight/obese women. Overweight/obese women who were minor allele carriers of rs2236212 [12] and rs3798713 had nominally lower DHA:DPAn3 index compared with major homozygotes. This led to nominally higher amounts of the substrate DPAn3. Lemaitre et al. found decreased DHA levels in the presence of at least one minor allele of rs2236212 [15], which is consistent with the tendency observed in our results. The normal-weight group generally showed the same trends. Moreover, among minor allele carriers of *ELOVL2* SNPs, overweight/obese women showed significantly higher DHA:EPA index than normal-weight women (DPA:EPA and DHA:DPA indexes showed the same tendency). This is in accordance with *FADS2* results. Therefore, minor alleles of *FADS2* and *ELOVL2* SNPs in overweight/obese women, could increase n-3 LC-PUFA production indexes, but without surpassing levels in normal-weight women.

Estrogen facilitates LC-PUFA synthesis [34, 35], probably by regulating delta-6 desaturase. During pregnancy, estrogen levels are higher, leading to increased amounts of DHA and AA until delivery, when the release of prolactin inhibits estrogen activity [35]. Since estrogen is produced in adipocytes, obesity is linked to high estrogen levels which increase proportionally to total body adiposity [36]. This could be an alternative explanation of the increase in n-3 LC-PUFA production observed in overweight/obese individuals. In this case, more studies introducing measurements of estrogen in both populations are needed.

We also studied n3:n6 ratios (EPA:AA and DHA:AA), which are associated with obesity risk. Among minor allele carriers of the *FADS* and *ELOVL* genes, normal-weight women showed nominally higher plasma ratios of EPA:AA and DHA:AA than women who were overweight/obese. The same trend was observed when comparing major homozygotes, but with a weaker (or no) association. This suggests that a high BMI leads to increased levels of AA and/or lower levels of DHA and EPA, more importantly in women who carry at least one copy of the minor allele of *FADS* and *ELOVL* SNPs.

To the best of our knowledge, this is the first report to directly associate *FADS* SNPs with obesity risk and to analyze how weight affects the impact of variations in genes involved in FA metabolism. Our results further justify the need for personalized nutrition by showing that metabolism is affected by nutritional status and genes. Our present study had some limitations: it might be limited by the relatively small sample size; however, we could identify and group participants into different weight and genotype categories. Information on dietary intake was obtained from validated food records and questionnaires and although each participant was guided by a nutritionist, this information could have been affected by recall bias. Likewise, supplementation data (brand, content, doses and frequency) during pregnancy were also obtained from questionnaires answered by the participants; therefore, this information might not be completely accurate.

## Conclusions

In conclusion, minor allele carriers of *FADS1* and *FADS2* SNPs have an increased risk of obesity (p≤0.05). The effects of genotype on FA concentrations differed by maternal pre-pregnancy weight status. Enzymatic activity and FA levels were reduced in normal-weight women who were minor allele carriers of *FADS* SNPs; these reductions were not significant in overweight/obese participants. This suggests that women with a BMI≥25 are less affected by *FADS* genetic variants in this regard. In the presence of *FADS2* and *ELOVL2* SNPs, overweight/obese women showed higher n-3 LC-PUFA production indexes than those women in the normal-weight group, but this was not enough to obtain a higher n-3 LC-PUFA concentration (p≤0.004). Since genotypes may not have the same effects on all people, it is of interest to continue exploring gene-BMI interactions to pursue personalized health-related recommendations. Alterations in maternal FAs modify the risk of pro-inflammatory diseases and affect FA delivery to the fetus/neonate, which has an impact on child growth and development. Therefore, this study also supports the importance of a healthy pre-pregnancy weight, and identifies groups of women who could benefit from a high intake of n-3 FAs in order to achieve an improved FA status that fulfills fetal/neonatal requirements.

## Supporting information

S1 Fig Metabolism pathways of omega-6 and omega-3 PUFAs. (TIF)

S1 Table DHA in plasma according to DHA supplementation. (DOCX)

S2 Table Characteristics of the studied SNPs within the *FADS* and *ELOVL* genes. (DOCX)

S3 Table Associations between plasma proportions of PUFAs and *FADS* and *ELOVL* polymorphisms. (DOCX)

S4 Table PUFA levels in plasma according to maternal *FADS* and *ELOVL* SNPs. (DOCX)

S5 Table Maternal EPA:AA and DHA:AA ratios in plasma according to their genotypes. (DOCX)
