# Supplementary figures and images for: Association of maternal weight with FADS and ELOVL genetic variants and fatty acid levels- The PREOBE follow-up
Source: PLoS One. 2017 Jun 9;12(6):e0179135. doi: 10.1371/journal.pone.0179135 (PMC5466308; doi:10.1371/journal.pone.0179135)

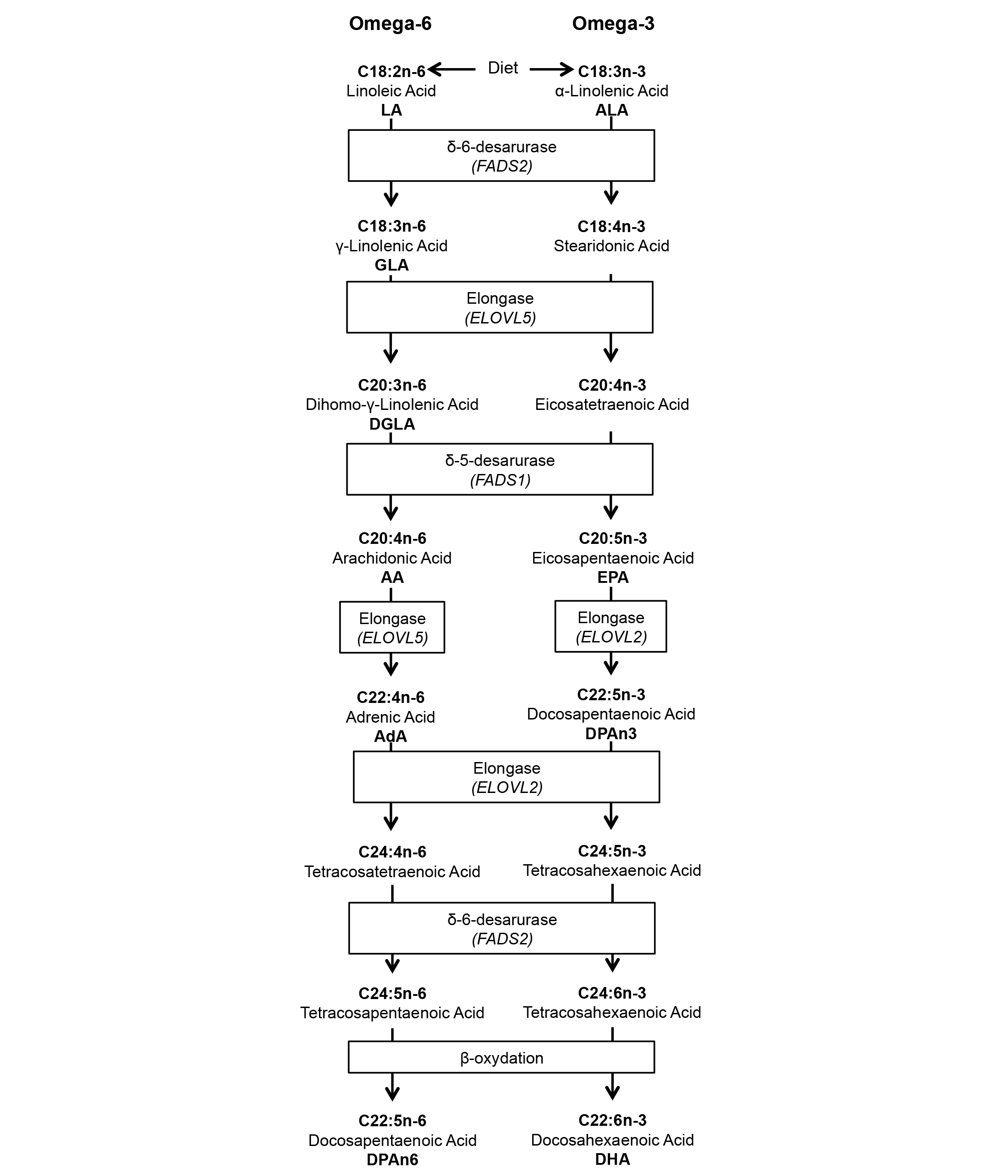

Supplement: S1 Fig — (TIF) [file pone.0179135.s001.tif]
