# Supplementary material for: Association of maternal weight with FADS and ELOVL genetic variants and fatty acid levels- The PREOBE follow-up
Source: PLoS One. 2017 Jun 9;12(6):e0179135. doi: 10.1371/journal.pone.0179135 (PMC5466308; doi:10.1371/journal.pone.0179135)
Supplement: S1 Table — (DOCX) [file pone.0179135.s002.docx]

S1 Table. DHA in plasma according to DHA supplementation.

|  | **DHA supplementation** | | **No DHA supplementation** | | **P** |
| --- | --- | --- | --- | --- | --- |
|  | **N** | **Mean % (SD)** | **N** | **Mean % (SD)** |  |
| **Total population (n= 110)** | 17 | 4.42 (0.88) | 93 | 4.28 (1.10) | 0.636 |
| **Normal-weight (n= 59)** | 11 | 4.21 (0.96) | 48 | 4.48 (1.21) | 0.485 |
| **Overweight/Obese (n= 51)** | 6 | 4.82 (0.57) | 45 | 4.09 (0.95) | 0.071 |
| * Indicates significant differences (p<0.05) after ANOVA test between groups of weight | | | | | |
| Abbreviations: DHA, docosahexaenoic acid. | | | |  |  |
