# Supplementary material for: Association of maternal weight with FADS and ELOVL genetic variants and fatty acid levels- The PREOBE follow-up
Source: PLoS One. 2017 Jun 9;12(6):e0179135. doi: 10.1371/journal.pone.0179135 (PMC5466308; doi:10.1371/journal.pone.0179135)
Supplement: S2 Table — (DOCX) [file pone.0179135.s003.docx]

**S2 Table.** Characteristics of the studied SNPs within the *FADS* and *ELOVL* genes.

a) *FADS* SNPs

| **Gene SNP** | ***Major/minor allele*** | **NORMAL-WEIGHT (n= 88)** | | | | **OVERWEIGHT/OBESE (n= 92)** | | | |
| --- | --- | --- | --- | --- | --- | --- | --- | --- | --- |
|  |  | **N** | **%** | **HWE*** | **Missing Analysis (%)** | **N** | **%** | **HWE*** | **Missing Analysis (%)** |
| ***FADS1*** |  |  |  |  |  |  |  |  |  |
| **rs174537** | ***G/T*** |  |  |  |  |  |  |  |  |
|  | ***MM*** | 22 | 46.80 | 0.313 | 46.59 | 18 | 36.00 | 0.263 | 45.65 |
|  | ***Mm*** | 18 | 38.30 |  |  | 27 | 54.00 |  |  |
|  | ***mm*** | 7 | 14.90 |  |  | 5 | 10.00 |  |  |
| **rs174545** | ***C/G*** |  |  |  |  |  |  |  |  |
|  | ***MM*** | 29 | 48.30 | 0.557 | 31.82 | 20 | 33.90 | 0.075 | 35.87 |
|  | ***Mm*** | 24 | 40.00 |  |  | 34 | 57.60 |  |  |
|  | ***mm*** | 7 | 11.70 |  |  | 5 | 8.50 |  |  |
| **rs174546** | ***C/T*** |  |  |  |  |  |  |  |  |
|  | ***MM*** | 30 | 49.20 | 0.518 | 30.68 | 20 | 32.80 | 0.045 | 33.70 |
|  | ***Mm*** | 24 | 39.30 |  |  | 36 | 59.00 |  |  |
|  | ***mm*** | 7 | 11.50 |  |  | 5 | 8.20 |  |  |
| **rs174548** | ***C/G*** |  |  |  |  |  |  |  |  |
|  | ***MM*** | 33 | 54.10 | 0.215 | 30.68 | 21 | 33.90 | 0.022 | 32.61 |
|  | ***Mm*** | 21 | 34.40 |  |  | 37 | 59.70 |  |  |
|  | ***mm*** | 7 | 11.50 |  |  | 4 | 6.50 |  |  |
| **rs174553** | ***A/G*** |  |  |  |  |  |  |  |  |
|  | ***MM*** | 30 | 49.20 | 0.518 | 30.68 | 20 | 32.30 | 0.350 | 32.61 |
|  | ***Mm*** | 24 | 39.30 |  |  | 37 | 59.70 |  |  |
|  | ***mm*** | 7 | 11.50 |  |  | 5 | 8.10 |  |  |
| **rs174561** | ***T/C*** |  |  |  |  |  |  |  |  |
|  | ***MM*** | 27 | 50.9% | 0.757 | 39.77 | 34 | 52.3% | 0.992 | 29.35 |
|  | ***Mm*** | 21 | 39.6% |  |  | 26 | 40.0% |  |  |
|  | ***mm*** | 5 | 9.4% |  |  | 5 | 7.7% |  |  |
| **rs174547** | ***T/C*** |  |  |  |  |  |  |  |  |
|  | ***MM*** | 22 | 44.90 | 0.777 | 44.32 | 17 | 37.00 | 0.219 | 50.00 |
|  | ***Mm*** | 21 | 42.90 |  |  | 25 | 54.30 |  |  |
|  | ***mm*** | 6 | 12.20 |  |  | 4 | 8.70 |  |  |
| ***FADS2*** |  |  |  |  |  |  |  |  |  |
| **rs1535** | ***A/G*** |  |  |  |  |  |  |  |  |
|  | ***MM*** | 28 | 49.10 | 0.420 | 35.23 | 20 | 35.10 | 0.119 | 38.04 |
|  | ***Mm*** | 22 | 38.60 |  |  | 32 | 56.10 |  |  |
|  | ***mm*** | 7 | 12.30 |  |  | 5 | 8.80 |  |  |
| **rs174575** | ***C/G*** |  |  |  |  |  |  |  |  |
|  | ***MM*** | 32 | 61.50 | 0.042 | 40.91 | 30 | 56.60 | 0.685 | 42.39 |
|  | ***Mm*** | 14 | 26.90 |  |  | 19 | 35.80 |  |  |
|  | ***mm*** | 6 | 11.50 |  |  | 4 | 7.50 |  |  |
| **rs174583** | ***C/T*** |  |  |  |  |  |  |  |  |
|  | ***MM*** | 26 | 45.60 | 0.434 | 35.23 | 19 | 31.70 | 0.083 | 34.78 |
|  | ***Mm*** | 23 | 40.40 |  |  | 35 | 58.30 |  |  |
|  | ***mm*** | 8 | 14.00 |  |  | 6 | 10.00 |  |  |
| **rs99780** | ***C/T*** |  |  |  |  |  |  |  |  |
|  | ***MM*** | 23 | 43.40 | 0.152 | 39.77 | 19 | 35.20 | 0.303 | 41.30 |
|  | ***Mm*** | 20 | 37.70 |  |  | 29 | 53.70 |  |  |
|  | ***mm*** | 10 | 18.90 |  |  | 6 | 11.10 |  |  |
| **rs174602** | ***T/C*** |  |  |  |  |  |  |  |  |
|  | ***MM***  ***Mm*** | 40  14 | 70.2%  24.6% | 0.254 | 35.23 | 39  22 | 60.0%  33.8% | 0.707 | 29.35 |
|  | ***mm*** | 3 | 5.3% |  |  | 4 | 6.2% |  |  |
| PREOBE cohort.  Major allele: M; minor allele: m  Data are expressed as percentages of total women in each weight group. | | | | | | | | | |
| *P-values of deviation from HWE among all subjects were tested by chi-square tests. | | | | | | | | | |

b) *ELOVL* SNPs

| **Gene SNP** | ***Major/minor allele*** | **NORMAL-WEIGHT (n= 88)** | | | | | **OVERWEIGHT/OBESE (n= 92)** | | | | | | | |
| --- | --- | --- | --- | --- | --- | --- | --- | --- | --- | --- | --- | --- | --- | --- |
|  |  | **N** | **%** | **HWE*** | | **Missing Analysis (%)** | **N** | | | **%** | | **HWE*** | | **Missing Analysis (%)** |
| ***ELOVL2*** |  |  | |  |  | | |  |  | |  | |  | |
| **rs2236212** | ***G/C*** |  |  |  |  | | |  |  | |  | |  | |
|  | ***MM*** | 16 | 29.10 | 0.923 | 37.50 | | | 22 | 36.70 | | 0.151 | | 34.78 | |
|  | ***Mm*** | 27 | 49.10 |  |  |  |  | 24 | 40.00 | |  |  |  |  |
|  | ***mm*** | 12 | 21.80 |  |  |  |  | 14 | 23.30 | |  |  |  |  |
| **rs3798713** | ***G/C*** |  |  |  |  | | |  |  | |  | |  | |
|  | ***MM*** | 13 | 24.50 | 0.506 | 39.77 | | | 19 | 32.20 | | 0.254 | | 35.87 | |
|  | ***Mm*** | 24 | 45.30 |  |  |  |  | 25 | 42.40 | |  |  |  |  |
|  | ***mm*** | 16 | 30.20 |  |  |  |  | 15 | 25.40 | |  |  |  |  |
| **rs953413** | ***A/G*** |  |  |  |  | | |  |  | |  | |  | |
|  | ***MM*** | 12 | 27.90 | 0.957 | 51.14 | | | 11 | 26.20 | | 0.227 | | 54.35 | |
|  | ***Mm*** | 18 | 41.90 |  |  |  |  | 17 | 40.50 | |  |  |  |  |
|  | ***mm*** | 13 | 30.20 |  |  |  |  | 14 | 33.30 | |  |  |  |  |
| ***ELOVL5*** |  |  | |  |  | | |  |  | |  | |  | |
| **rs2397142** | ***C/G*** |  |  |  |  | | |  |  | |  | |  | |
|  | ***MM*** | 28 | 48.30 | 0.506 | 34.09 | | | 29 | 50.00 | | 0.205 | | 36.96 | |
|  | ***Mm*** | 23 | 39.70 |  |  |  |  | 21 | 36.20 | |  |  |  |  |
|  | ***mm*** | 7 | 12.10 |  |  |  |  | 8 | 13.80 | |  |  |  |  |
| **rs9395855** | ***T/G*** |  |  |  |  | | |  |  | |  | |  | |
|  | ***MM***  ***Mm*** | 9  18 | 23.10  46.20 | 0.656 | 55.68 | | | 11  19 | 29.70  51.40 | | 0.812 | | 59.78 | |
|  | ***mm*** | 12 | 30.80 |  |  |  |  | 7 | 18.90 | |  |  |  |  |
| PREOBE cohort.  Major allele: M; minor allele: m  Data are expressed as percentages of total women in each weight group. | | | | | | | | | | | | | | |
| *P-values of deviation from HWE among all subjects were tested by chi-square tests. | | | | | | | | | | | | | | |
