# Supplementary material for: Association of maternal weight with FADS and ELOVL genetic variants and fatty acid levels- The PREOBE follow-up
Source: PLoS One. 2017 Jun 9;12(6):e0179135. doi: 10.1371/journal.pone.0179135 (PMC5466308; doi:10.1371/journal.pone.0179135)
Supplement: S3 Table — (DOCX) [file pone.0179135.s004.docx]

**S3 Table.** Associations between plasma proportions of PUFAs and *FADS* and *ELOVL* polymorphisms.

a) *FADS1* indexes

| **Fatty acid** | **Gene** | **SNP *Major/minor allele*** | | **NORMAL-WEIGHT** | | | **OVERWEIGHT/OBESE** | | |
| --- | --- | --- | --- | --- | --- | --- | --- | --- | --- |
|  |  |  |  | **N** | **β** | **P** | **N** | **β** | **P** |
| ***FADS1* indexes** | | | | | | | | | |
| **AA:LA** |  |  |  |  |  |  |  |  |  |
|  | *FADS1* | rs174537 | *G/T* | 22 | -0.23 | 0.498 | 18 | -0.03 | 0.935 |
|  | *FADS1* | rs174545 | *C/G* | 28 | -0.39 | 0.171 | 17 | -0.05 | 0.865 |
|  | *FADS1* | rs174546 | *C/T* | 28 | -0.39 | 0.171 | 19 | -0.04 | 0.890 |
|  | *FADS1* | rs174548 | *C/G* | 28 | -0.21 | 0.361 | 19 | -0.04 | 0.890 |
|  | *FADS1* | rs174553 | *A/G* | 28 | -0.39 | 0.171 | 19 | -0.04 | 0.890 |
|  | *FADS1* | rs174561 | *T/C* | 9 | -0.20 | 0.703 | 11 | 0.35 | 0.397 |
|  | *FADS1* | rs174547 | *T/C* | 23 | -0.34 | 0.289 | 18 | -0.03 | 0.935 |
| **AA:DGLA** |  |  |  |  |  |  |  |  |  |
|  | *FADS1* | rs174537 | *G/T* | 22 | -0.81 | **0.004*** | 18 | -0.48 | 0.155 |
|  | *FADS1* | rs174545 | *C/G* | 28 | -0.70 | **0.003*** | 17 | -0.48 | 0.174 |
|  | *FADS1* | rs174546 | *C/T* | 28 | -0.70 | **0.003*** | 19 | -0.49 | 0.139 |
|  | *FADS1* | rs174548 | *C/G* | 28 | -0.36 | 0.071 | 19 | -0.49 | 0.139 |
|  | *FADS1* | rs174553 | *A/G* | 28 | -0.70 | **0.003*** | 19 | -0.49 | 0.139 |
|  | *FADS1* | rs174561 | *T/C* | 9 | -0.21 | 0.655 | 11 | -0.22 | 0.692 |
|  | *FADS1* | rs174547 | *T/C* | 23 | -0.67 | **0.013** | 18 | -0.48 | 0.155 |
| **EPA:ALA** |  |  |  |  |  |  |  |  |  |
|  | *FADS1* | rs174537 | *G/T* | 22 | -0.30 | 0.343 | 17 | 0.12 | 0.721 |
|  | *FADS1* | rs174545 | *C/G* | 28 | -0.35 | 0.183 | 16 | 0.12 | 0.733 |
|  | *FADS1* | rs174546 | *C/T* | 28 | -0.35 | 0.183 | 18 | 0.10 | 0.761 |
|  | *FADS1* | rs174548 | *C/G* | 28 | 0.06 | 0.778 | 18 | 0.10 | 0.761 |
|  | *FADS1* | rs174553 | *A/G* | 28 | -0.35 | 0.183 | 18 | 0.10 | 0.761 |
|  | *FADS1* | rs174561 | *T/C* | 9 | -0.45 | 0.318 | 11 | 0.25 | 0.597 |
|  | *FADS1* | rs174547 | *T/C* | 23 | -0.35 | 0.236 | 17 | 0.12 | 0.721 |
| Associations between SNPs and fatty acids were analyzed using linear regression. SNPs were coded according to minor allele count and analyzed as a numeric variable. "β"= beta per minor allele standardized per the major allele. All associations were adjusted for potential confounders such as age, education, smoking and energy intake. P-values <0.05 are highlighted in bold and significant associations that persisted after Bonferroni correction are additionally denoted by stars (*p<0.004). LA: Linoleic Acid; GLA: γ-Linolenic Acid; DGLA: Dihomo-γ-Linolenic Acid; AA: Arachidonic Acid; AdA: Adrenic Acid; DPAn6: Docosapentaenoic acid n6; ALA: α-linolenic Acid; EPA: Eicosapentaenoic acid; DPAn3: Docosapentaenoic acid n3; DHA: Docosahexaenoic acid. | | | | | | | | | |
|  |  |  |  |  |  |  |  |  |  |
|  |  |  |  |  |  |  |  |  |  |
|  |  |  |  |  |  |  |  |  |  |
|  |  |  |  |  |  |  |  |  |  |
|  |  |  |  |  |  |  |  |  |  |
|  |  |  |  |  |  |  |  |  |  |

b) *FADS2* indexes

| **Fatty acid** | **Gene** | **SNP *Major/minor allele*** | | **NORMAL-WEIGHT** | | | **OVERWEIGHT/OBESE** | | |
| --- | --- | --- | --- | --- | --- | --- | --- | --- | --- |
|  |  |  |  | **N** | **β** | **P** | **N** | **β** | **P** |
| ***FADS2* indexes** | | | | | | | | | |
| **GLA:LA** |  |  |  |  |  |  |  |  |  |
|  | *FADS2* | rs1535 | *A/G* | 25 | 0.01 | 0.984 | 18 | -0.10 | 0.761 |
|  | *FADS2* | rs174575 | *C/G* | 22 | 0.20 | 0.451 | 19 | 0.59 | 0.054 |
|  | *FADS2* | rs174583 | *C/T* | 26 | -0.04 | 0.891 | 19 | -0.13 | 0.699 |
|  | *FADS2* | rs99780 | *C/T* | 24 | -0.08 | 0.786 | 18 | -0.10 | 0.761 |
|  | *FADS2* | rs174602 | *T/C* | 11 | 0.23 | 0.630 | 11 | -0.50 | 0.372 |
| **DGLA:LA** |  |  |  |  |  |  |  |  |  |
|  | *FADS2* | rs1535 | *A/G* | 26 | 0.56 | **0.029** | 18 | 0.42 | 0.226 |
|  | *FADS2* | rs174575 | *C/G* | 23 | 0.51 | 0.062 | 19 | -0.05 | 0.889 |
|  | *FADS2* | rs174583 | *C/T* | 27 | 0.53 | **0.040** | 19 | 0.41 | 0.226 |
|  | *FADS2* | rs99780 | *C/T* | 25 | 0.54 | **0.042** | 18 | 0.42 | 0.226 |
|  | *FADS2* | rs174602 | *T/C* | 11 | 0.38 | 0.181 | 11 | 0.44 | 0.489 |
| **AA:LA** |  |  |  |  |  |  |  |  |  |
|  | *FADS2* | rs1535 | *A/G* | 26 | -0.32 | 0.271 | 18 | -0.03 | 0.935 |
|  | *FADS2* | rs174575 | *C/G* | 23 | -0.03 | 0.909 | 19 | 0.11 | 0.720 |
|  | *FADS2* | rs174583 | *C/T* | 27 | -0.35 | 0.218 | 19 | -0.04 | 0.890 |
|  | *FADS2* | rs99780 | *C/T* | 25 | -0.06 | 0.849 | 18 | -0.03 | 0.935 |
|  | *FADS2* | rs174602 | *T/C* | 11 | 0.15 | 0.772 | 11 | -0.51 | 0.335 |
| **DPAn6:AA** |  |  |  |  |  |  |  |  |  |
|  | *FADS2* | rs1535 | *A/G* | 26 | 0.27 | 0.271 | 18 | 0.18 | 0.484 |
|  | *FADS2* | rs174575 | *C/G* | 23 | -0.05 | 0.836 | 19 | -0.07 | 0.772 |
|  | *FADS2* | rs174583 | *C/T* | 27 | 0.29 | 0.236 | 19 | 0.18 | 0.466 |
|  | *FADS2* | rs99780 | *C/T* | 25 | 0.01 | 0.981 | 18 | 0.18 | 0.484 |
|  | *FADS2* | rs174602 | *T/C* | 11 | 0.46 | 0.124 | 11 | 0.02 | 0.958 |
| **DPAn6:AdA** |  |  |  |  |  |  |  |  |  |
|  | *FADS2* | rs1535 | *A/G* | 26 | 0.04 | 0.873 | 17 | 0.27 | 0.336 |
|  | *FADS2* | rs174575 | *C/G* | 23 | -0.15 | 0.579 | 18 | -0.26 | 0.333 |
|  | *FADS2* | rs174583 | *C/T* | 27 | 0.03 | 0.916 | 18 | 0.28 | 0.304 |
|  | *FADS2* | rs99780 | *C/T* | 25 | -0.42 | 0.094 | 17 | 0.27 | 0.336 |
|  | *FADS2* | rs174602 | *T/C* | 11 | -0.10 | 0.792 | 10 | 0.52 | 0.318 |
| **EPA:ALA** |  |  |  |  |  |  |  |  |  |
|  | *FADS2* | rs1535 | *A/G* | 26 | -0.33 | 0.228 | 17 | 0.12 | 0.721 |
|  | *FADS2* | rs174575 | *C/G* | 23 | -0.19 | 0.484 | 18 | 0.35 | 0.254 |
|  | *FADS2* | rs174583 | *C/T* | 27 | -0.33 | 0.216 | 18 | 0.10 | 0.761 |
|  | *FADS2* | rs99780 | *C/T* | 25 | 0.26 | 0.338 | 17 | 0.12 | 0.721 |
|  | *FADS2* | rs174602 | *T/C* | 11 | -0.13 | 0.739 | 11 | 0.34 | 0.566 |
| **DHA:EPA** |  |  |  |  |  |  |  |  |  |
|  | *FADS2* | rs1535 | *A/G* | 26 | 0.20 | 0.486 | 17 | -0.10 | 0.703 |
|  | *FADS2* | rs174575 | *C/G* | 23 | -0.13 | 0.665 | 18 | -0.23 | 0.375 |
|  | *FADS2* | rs174583 | *C/T* | 27 | 0.19 | 0.497 | 18 | -0.08 | 0.762 |
|  | *FADS2* | rs99780 | *C/T* | 25 | -0.29 | 0.276 | 17 | -0.10 | 0.703 |
|  | *FADS2* | rs174602 | *T/C* | 11 | 0.23 | 0.556 | 11 | -0.47 | 0.388 |
| **DHA:DPAn3** |  |  |  |  |  |  |  |  |  |
|  | *FADS2* | rs1535 | *A/G* | 26 | 0.12 | 0.658 | 18 | 0.34 | 0.273 |
|  | *FADS2* | rs174575 | *C/G* | 23 | 0.00 | 0.995 | 19 | 0.12 | 0.695 |
|  | *FADS2* | rs174583 | *C/T* | 27 | 0.14 | 0.617 | 19 | 0.35 | 0.251 |
|  | *FADS2* | rs99780 | *C/T* | 25 | -0.13 | 0.641 | 18 | 0.34 | 0.273 |
|  | *FADS2* | rs174602 | *T/C* | 11 | -0.52 | 0.164 | 11 | -0.52 | 0.312 |
| Associations between SNPs and fatty acids were analyzed using linear regression. SNPs were coded according to minor allele count and analyzed as a numeric variable. "β"= beta per minor allele standardized per the major allele. All associations were adjusted for potential confounders such as age, education, smoking and energy intake. P-values <0.05 are highlighted in bold and significant associations that persisted after Bonferroni correction are additionally denoted by stars (*p<0.004). LA: Linoleic Acid; GLA: γ-Linolenic Acid; DGLA: Dihomo-γ-Linolenic Acid; AA: Arachidonic Acid; AdA: Adrenic Acid; DPAn6: Docosapentaenoic acid n6; ALA: α-linolenic Acid; EPA: Eicosapentaenoic acid; DPAn3: Docosapentaenoic acid n3; DHA: Docosahexaenoic acid. | | | | | | | | | |
|  |  |  |  |  |  |  |  |  |  |
|  |  |  |  |  |  |  |  |  |  |
|  |  |  |  |  |  |  |  |  |  |
|  |  |  |  |  |  |  |  |  |  |
|  |  |  |  |  |  |  |  |  |  |
|  |  |  |  |  |  |  |  |  |  |

c) *ELOVL* indexes

| **Fatty acid** | **Gene** | **SNP *Major/minor allele*** | | **NORMAL-WEIGHT** | | | **OVERWEIGHT/OBESE** | | |
| --- | --- | --- | --- | --- | --- | --- | --- | --- | --- |
|  |  |  |  | **N** | **β** | **P** | **N** | **β** | **P** |
| ***ELOVL2* indexes** | | | | | | | | | |
| **DPAn6:AdA** |  |  |  |  |  |  |  |  |  |
|  | *ELOVL2* | rs2236212 | *G/C* | 25 | 0.07 | 0.744 | 18 | -0.19 | 0.427 |
|  | *ELOVL2* | rs3798713 | *G/C* | 25 | 0.12 | 0.579 | 18 | -0.19 | 0.427 |
|  | *ELOVL2* | rs953413 | *A/G* | 21 | -0.01 | 0.978 | 16 | -0.04 | 0.887 |
| **DPAn3:EPA** |  |  |  |  |  |  |  |  |  |
|  | *ELOVL2* | rs2236212 | *G/C* | 25 | 0.05 | 0.822 | 18 | 0.00 | 0.987 |
|  | *ELOVL2* | rs3798713 | *G/C* | 25 | 0.09 | 0.679 | 18 | 0.00 | 0.987 |
|  | *ELOVL2* | rs953413 | *A/G* | 21 | 0.14 | 0.554 | 16 | 0.30 | 0.115 |
| **DHA:EPA** |  |  |  |  |  |  |  |  |  |
|  | *ELOVL2* | rs2236212 | *G/C* | 25 | -0.07 | 0.750 | 18 | -0.12 | 0.642 |
|  | *ELOVL2* | rs3798713 | *G/C* | 25 | 0.01 | 0.953 | 18 | -0.12 | 0.642 |
|  | *ELOVL2* | rs953413 | *A/G* | 21 | 0.04 | 0.871 | 16 | 0.24 | 0.271 |
| **DHA:DPAn3** |  |  |  |  |  |  |  |  |  |
|  | *ELOVL2* | rs2236212 | *G/C* | 25 | -0.05 | 0.806 | 19 | -0.58 | **0.016** |
|  | *ELOVL2* | rs3798713 | *G/C* | 25 | 0.00 | 0.995 | 19 | -0.58 | **0.016** |
|  | *ELOVL2* | rs953413 | *A/G* | 21 | -0.21 | 0.402 | 17 | -0.06 | 0.834 |
| ***ELOVL5* indexes** | | | | | | | | | |
| **DGLA:LA** |  |  |  |  |  |  |  |  |  |
|  | *ELOVL5* | rs2397142 | *C/G* | 27 | -0.33 | 0.153 | 19.00 | -0.03 | 0.923 |
|  | *ELOVL5* | rs9395855 | *T/G* | 19 | 0.00 | 0.988 | 17.00 | 0.20 | 0.507 |
| **DGLA:GLA** |  |  |  |  |  |  |  |  |  |
|  | *ELOVL5* | rs2397142 | *C/G* | 26 | -0.12 | 0.637 | 19.00 | -0.01 | 0.977 |
|  | *ELOVL5* | rs9395855 | *T/G* | 18 | -0.18 | 0.490 | 17.00 | -0.10 | 0.745 |
| **AA:LA** |  |  |  |  |  |  |  |  |  |
|  | *ELOVL5* | rs2397142 | *C/G* | 27 | -0.11 | 0.644 | 19.00 | -0.24 | 0.330 |
|  | *ELOVL5* | rs9395855 | *T/G* | 19 | 0.30 | 0.289 | 17.00 | 0.35 | 0.155 |
| **AdA:AA** |  |  |  |  |  |  |  |  |  |
|  | *ELOVL5* | rs2397142 | *C/G* | 27 | -0.03 | 0.903 | 18.00 | 0.08 | 0.726 |
|  | *ELOVL5* | rs9395855 | *T/G* | 19 | -0.20 | 0.422 | 16.00 | -0.06 | 0.800 |
| **EPA:ALA** |  |  |  |  |  |  |  |  |  |
|  | *ELOVL5* | rs2397142 | *C/G* | 27 | 0.11 | 0.634 | 18 | 0.35 | 0.182 |
|  | *ELOVL5* | rs9395855 | *T/G* | 19 | 0.09 | 0.757 | 16 | -0.39 | 0.124 |
| Associations between SNPs and fatty acids were analyzed using linear regression. SNPs were coded according to minor allele count and analyzed as a numeric variable. "β"= beta per minor allele standardized per the major allele. All associations were adjusted for potential confounders such as age, education, smoking and energy intake. P-values <0.05 are highlighted in bold and significant associations that persisted after Bonferroni correction are additionally denoted by stars (*p<0.004). LA: Linoleic Acid; GLA: γ-Linolenic Acid; DGLA: Dihomo-γ-Linolenic Acid; AA: Arachidonic Acid; AdA: Adrenic Acid; DPAn6: Docosapentaenoic acid n6; ALA: α-linolenic Acid; EPA: Eicosapentaenoic acid; DPAn3: Docosapentaenoic acid n3; DHA: Docosahexaenoic acid. | | | | | | | | | |
|  |  |  |  |  |  |  |  |  |  |
|  |  |  |  |  |  |  |  |  |  |
|  |  |  |  |  |  |  |  |  |  |
|  |  |  |  |  |  |  |  |  |  |
|  |  |  |  |  |  |  |  |  |  |
|  |  |  |  |  |  |  |  |  |  |

d) Fatty acids involved in *FADS1* indexes

| **Fatty acid** | **Gene** | **SNP *Major/minor allele*** | | **NORMAL-WEIGHT** | | | **OVERWEIGHT/OBESE** | | |
| --- | --- | --- | --- | --- | --- | --- | --- | --- | --- |
|  |  |  |  | **N** | **β** | **P** | **N** | **β** | **P** |
| **Fatty acids involved in *FADS1* indexes** | | |  |  |  |  |  |  |  |
| **C18:2n-6 (LA)** |  |  |  |  |  |  |  |  |  |
|  | *FADS1* | rs174537 | *G/T* | 22 | -0.37 | 0.263 | 18 | 0.01 | 0.978 |
|  | *FADS1* | rs174545 | *C/G* | 28 | -0.04 | 0.888 | 17 | 0.02 | 0.948 |
|  | *FADS1* | rs174546 | *C/T* | 28 | -0.04 | 0.888 | 19 | 0.01 | 0.965 |
|  | *FADS1* | rs174548 | *C/G* | 28 | -0.13 | 0.573 | 19 | 0.01 | 0.965 |
|  | *FADS1* | rs174553 | *A/G* | 28 | -0.04 | 0.888 | 19 | 0.01 | 0.965 |
|  | *FADS1* | rs174561 | *T/C* | 9 | 0.14 | 0.763 | 11 | -0.31 | 0.507 |
|  | *FADS1* | rs174547 | *T/C* | 23 | -0.20 | 0.370 | 18 | 0.01 | 0.978 |
| **C20:3n-6 (DGLA)** | |  |  |  |  |  |  |  |  |
|  | *FADS1* | rs174537 | *G/T* | 22 | 0.75 | **0.012** | 18 | 0.53 | 0.119 |
|  | *FADS1* | rs174545 | *C/G* | 28 | 0.57 | **0.028** | 17 | 0.52 | 0.137 |
|  | *FADS1* | rs174546 | *C/T* | 28 | 0.57 | **0.028** | 19 | 0.52 | 0.118 |
|  | *FADS1* | rs174548 | *C/G* | 28 | 0.34 | 0.109 | 19 | 0.52 | 0.118 |
|  | *FADS1* | rs174553 | *A/G* | 28 | 0.57 | **0.028** | 19 | 0.52 | 0.118 |
|  | *FADS1* | rs174561 | *T/C* | 9 | 0.11 | 0.495 | 11 | 0.36 | 0.503 |
|  | *FADS1* | rs174547 | *T/C* | 23 | 0.60 | **0.034** | 18 | 0.53 | 0.119 |
| **C20:4n-6 (AA)** |  |  |  |  |  |  |  |  |  |
|  | *FADS1* | rs174537 | *G/T* | 22 | -0.57 | 0.092 | 18 | 0.01 | 0.984 |
|  | *FADS1* | rs174545 | *C/G* | 28 | -0.55 | **0.042** | 17 | -0.01 | 0.977 |
|  | *FADS1* | rs174546 | *C/T* | 28 | -0.55 | **0.042** | 19 | -0.01 | 0.971 |
|  | *FADS1* | rs174548 | *C/G* | 28 | -0.36 | 0.102 | 19 | -0.01 | 0.971 |
|  | *FADS1* | rs174553 | *A/G* | 28 | -0.55 | **0.042** | 19 | -0.01 | 0.971 |
|  | *FADS1* | rs174561 | *T/C* | 9 | -0.16 | 0.793 | 11 | 0.18 | 0.666 |
|  | *FADS1* | rs174547 | *T/C* | 23 | -0.53 | 0.087 | 18 | 0.01 | 0.984 |
| **C18:3n-3 (ALA)** | | | | | | | | | |
|  | *FADS1* | rs174537 | *G/T* | 22 | 0.71 | **0.027** | 18 | -0.09 | 0.790 |
|  | *FADS1* | rs174545 | *C/G* | 28 | 0.47 | 0.084 | 17 | -0.08 | 0.814 |
|  | *FADS1* | rs174546 | *C/T* | 28 | 0.47 | 0.084 | 19 | -0.09 | 0.774 |
|  | *FADS1* | rs174548 | *C/G* | 28 | 0.17 | 0.436 | 19 | -0.09 | 0.774 |
|  | *FADS1* | rs174553 | *A/G* | 28 | 0.47 | 0.084 | 19 | -0.09 | 0.774 |
|  | *FADS1* | rs174561 | *T/C* | 9 | 0.25 | 0.557 | 11 | -0.57 | 0.081 |
|  | *FADS1* | rs174547 | *T/C* | 23 | 0.66 | **0.026** | 18 | -0.09 | 0.790 |
| **C20:5n3 (EPA)** |  |  |  |  |  |  |  |  |  |
|  | *FADS1* | rs174537 | *G/T* | 22 | 0.21 | 0.478 | 17 | 0.11 | 0.749 |
|  | *FADS1* | rs174545 | *C/G* | 28 | -0.05 | 0.856 | 16 | 0.11 | 0.751 |
|  | *FADS1* | rs174546 | *C/T* | 28 | -0.05 | 0.856 | 18 | 0.10 | 0.780 |
|  | *FADS1* | rs174548 | *C/G* | 28 | 0.22 | 0.285 | 18 | 0.10 | 0.780 |
|  | *FADS1* | rs174553 | *A/G* | 28 | -0.05 | 0.856 | 18 | 0.10 | 0.780 |
|  | *FADS1* | rs174561 | *T/C* | 9 | -0.26 | 0.559 | 11 | -0.03 | 0.943 |
|  | *FADS1* | rs174547 | *T/C* | 23 | 0.05 | 0.866 | 17 | 0.11 | 0.749 |
| Associations between SNPs and fatty acids were analyzed using linear regression. SNPs were coded according to minor allele count and analyzed as a numeric variable. "β"= beta per minor allele standardized per the major allele. All associations were adjusted for potential confounders such as age, education, smoking and energy intake. P-values <0.05 are highlighted in bold and significant associations that persisted after Bonferroni correction are additionally denoted by stars (*p<0.004). LA: Linoleic Acid; GLA: γ-Linolenic Acid; DGLA: Dihomo-γ-Linolenic Acid; AA: Arachidonic Acid; AdA: Adrenic Acid; DPAn6: Docosapentaenoic acid n6; ALA: α-linolenic Acid; EPA: Eicosapentaenoic acid; DPAn3: Docosapentaenoic acid n3; DHA: Docosahexaenoic acid. | | | | | | | | | |
|  |  |  |  |  |  |  |  |  |  |
|  |  |  |  |  |  |  |  |  |  |
|  |  |  |  |  |  |  |  |  |  |
|  |  |  |  |  |  |  |  |  |  |
|  |  |  |  |  |  |  |  |  |  |
|  |  |  |  |  |  |  |  |  |  |

e) Fatty acids involved in *FADS2* indexes

| **Fatty acid** | **Gene** | **SNP *Major/minor allele*** | | **NORMAL-WEIGHT** | | | **OVERWEIGHT/OBESE** | | |
| --- | --- | --- | --- | --- | --- | --- | --- | --- | --- |
|  |  |  |  | **N** | **β** | **P** | **N** | **β** | **P** |
| **Fatty acids involved in *FADS2* indexes** | | |  |  |  |  |  |  |  |
| **C18:2n-6 (LA)** |  |  |  |  |  |  |  |  |  |
|  | *FADS2* | rs1535 | *A/G* | 26 | -0.10 | 0.728 | 18 | 0.01 | 0.978 |
|  | *FADS2* | rs174575 | *C/G* | 23 | -0.24 | 0.424 | 19 | -0.14 | 0.669 |
|  | *FADS2* | rs174583 | *C/T* | 27 | -0.09 | 0.763 | 19 | 0.01 | 0.965 |
|  | *FADS2* | rs99780 | *C/T* | 25 | -0.48 | 0.085 | 18 | 0.01 | 0.978 |
|  | *FADS2* | rs174602 | *T/C* | 11 | -0.39 | 0.446 | 11 | 0.21 | 0.734 |
| **C18:3n6 (GLA)** |  |  |  |  |  |  |  |  |  |
|  | *FADS2* | rs1535 | *A/G* | 25 | -0.07 | 0.811 | 18 | -0.08 | 0.806 |
|  | *FADS2* | rs174575 | *C/G* | 22 | 0.12 | 0.672 | 19 | 0.64 | **0.031** |
|  | *FADS2* | rs174583 | *C/T* | 26 | -0.12 | 0.695 | 19 | -0.11 | 0.740 |
|  | *FADS2* | rs99780 | *C/T* | 24 | -0.24 | 0.429 | 18 | -0.08 | 0.806 |
|  | *FADS2* | rs174602 | *T/C* | 11 | 0.19 | 0.690 | 11 | -0.48 | 0.327 |
| **C20:3n-6 (DGLA)** | |  |  |  |  |  |  |  |  |
|  | *FADS2* | rs1535 | *A/G* | 26 | 0.65 | **0.010** | 18 | 0.53 | 0.119 |
|  | *FADS2* | rs174575 | *C/T* | 23 | 0.51 | 0.054 | 19 | -0.11 | 0.750 |
|  | *FADS2* | rs174583 | *C/G* | 27 | 0.62 | **0.015** | 19 | 0.52 | 0.118 |
|  | *FADS2* | rs99780 | *C/T* | 25 | 0.43 | 0.109 | 18 | 0.53 | 0.119 |
|  | *FADS2* | rs174602 | *T/C* | 11 | 0.33 | 0.065 | 11 | 0.77 | 0.230 |
| **C20:4n-6 (AA)** |  |  |  |  |  |  |  |  |  |
|  | *FADS2* | rs1535 | *A/G* | 26 | -0.50 | 0.073 | 18 | 0.01 | 0.984 |
|  | *FADS2* | rs174575 | *C/T* | 23 | -0.24 | 0.396 | 19 | 0.06 | 0.838 |
|  | *FADS2* | rs174583 | *C/G* | 27 | -0.52 | 0.054 | 19 | -0.01 | 0.971 |
|  | *FADS2* | rs99780 | *C/T* | 25 | -0.41 | 0.155 | 18 | 0.01 | 0.984 |
|  | *FADS2* | rs174602 | *T/C* | 11 | -0.03 | 0.957 | 11 | -0.51 | 0.319 |
| **C22:4n-6 (AdA)** | | | | | | | | | |
|  | *FADS2* | rs1535 | *A/G* | 26 | -0.04 | 0.882 | 17 | -0.01 | 0.965 |
|  | *FADS2* | rs174575 | *C/G* | 23 | -0.07 | 0.819 | 18 | -0.13 | 0.616 |
|  | *FADS2* | rs174583 | *C/T* | 27 | -0.04 | 0.873 | 18 | -0.03 | 0.906 |
|  | *FADS2* | rs99780 | *C/T* | 25 | 0.12 | 0.678 | 17 | -0.01 | 0.965 |
|  | *FADS2* | rs174602 | *T/C* | 11 | 0.61 | 0.138 | 10 | 0.02 | 0.964 |
| **C22:5n-6 (DPAn6)** | |  |  |  |  |  |  |  |  |
|  | *FADS2* | rs1535 | *A/G* | 26 | 0.01 | 0.973 | 18 | 0.21 | 0.341 |
|  | *FADS2* | rs174575 | *C/G* | 23 | -0.15 | 0.578 | 19 | -0.07 | 0.732 |
|  | *FADS2* | rs174583 | *C/T* | 27 | 0.00 | 0.999 | 19 | 0.20 | 0.339 |
|  | *FADS2* | rs99780 | *C/T* | 25 | -0.17 | 0.501 | 18 | 0.21 | 0.341 |
|  | *FADS2* | rs174602 | *T/C* | 11 | 0.42 | 0.095 | 11 | -0.11 | 0.768 |
| **C18:3n-3 (ALA)** | | | | | | | | | |
|  | *FADS2* | rs1535 | *A/G* | 26 | 0.45 | 0.105 | 18 | -0.09 | 0.790 |
|  | *FADS2* | rs174575 | *C/G* | 23 | 0.50 | **0.043** | 19 | 0.22 | 0.494 |
|  | *FADS2* | rs174583 | *C/T* | 27 | 0.44 | 0.106 | 19 | -0.09 | 0.774 |
|  | *FADS2* | rs99780 | *C/T* | 25 | 0.32 | 0.270 | 18 | -0.09 | 0.790 |
|  | *FADS2* | rs174602 | *T/C* | 11 | -0.38 | 0.397 | 11 | -0.15 | 0.753 |
| **C20:5n3 (EPA)** |  |  |  |  |  |  |  |  |  |
|  | *FADS2* | rs1535 | *A/G* | 26 | -0.04 | 0.887 | 17 | 0.11 | 0.749 |
|  | *FADS2* | rs174575 | *C/G* | 23 | 0.12 | 0.671 | 18 | 0.37 | 0.252 |
|  | *FADS2* | rs174583 | *C/T* | 27 | -0.05 | 0.856 | 18 | 0.10 | 0.780 |
|  | *FADS2* | rs99780 | *C/T* | 25 | 0.52 | **0.035** | 17 | 0.11 | 0.749 |
|  | *FADS2* | rs174602 | *T/C* | 11 | -0.17 | 0.622 | 11 | 0.39 | 0.494 |
| **C22:5n-3 (DPAn3)** | |  |  |  |  |  |  |  |  |
|  | *FADS2* | rs1535 | *A/G* | 26 | -0.03 | 0.916 | 18 | -0.41 | 0.173 |
|  | *FADS2* | rs174575 | *C/G* | 23 | 0.01 | 0.969 | 19 | 0.05 | 0.867 |
|  | *FADS2* | rs174583 | *C/T* | 27 | -0.06 | 0.825 | 19 | -0.42 | 0.153 |
|  | *FADS2* | rs99780 | *C/T* | 25 | 0.62 | **0.011** | 18 | -0.41 | 0.173 |
|  | *FADS2* | rs174602 | *T/C* | 11 | 0.22 | 0.517 | 11 | 0.33 | 0.467 |
| **C22:6n-3 (DHA)** | | | | | | | | | |
|  | *FADS2* | rs1535 | *A/G* | 26 | 0.08 | 0.761 | 18 | -0.16 | 0.563 |
|  | *FADS2* | rs174575 | *C/G* | 23 | 0.01 | 0.974 | 19 | 0.24 | 0.365 |
|  | *FADS2* | rs174583 | *C/T* | 27 | 0.07 | 0.795 | 19 | -0.17 | 0.522 |
|  | *FADS2* | rs99780 | *C/T* | 25 | 0.42 | 0.106 | 18 | -0.16 | 0.563 |
|  | *FADS2* | rs174602 | *T/C* | 11 | -0.32 | 0.403 | 11 | -0.24 | 0.620 |
| Associations between SNPs and fatty acids were analyzed using linear regression. SNPs were coded according to minor allele count and analyzed as a numeric variable. "β"= beta per minor allele standardized per the major allele. All associations were adjusted for potential confounders such as age, education, smoking and energy intake. P-values <0.05 are highlighted in bold and significant associations that persisted after Bonferroni correction are additionally denoted by stars (*p<0.004). LA: Linoleic Acid; GLA: γ-Linolenic Acid; DGLA: Dihomo-γ-Linolenic Acid; AA: Arachidonic Acid; AdA: Adrenic Acid; DPAn6: Docosapentaenoic acid n6; ALA: α-linolenic Acid; EPA: Eicosapentaenoic acid; DPAn3: Docosapentaenoic acid n3; DHA: Docosahexaenoic acid. | | | | | | | | | |
|  |  |  |  |  |  |  |  |  |  |
|  |  |  |  |  |  |  |  |  |  |
|  |  |  |  |  |  |  |  |  |  |
|  |  |  |  |  |  |  |  |  |  |
|  |  |  |  |  |  |  |  |  |  |
|  |  |  |  |  |  |  |  |  |  |

f) Fatty acids involved in *ELOVL* indexes

| **Fatty acid** | **Gene** | **SNP *Major/minor allele*** | | **NORMAL-WEIGHT** | | | **OVERWEIGHT/OBESE** | | |
| --- | --- | --- | --- | --- | --- | --- | --- | --- | --- |
|  |  |  |  | **N** | **β** | **P** | **N** | **β** | **P** |
| **Fatty acids involved in *ELOVL2* indexes** | | |  |  |  |  |  |  |  |
| **C22:4n-6 (AdA)** | | | | | | | | | |
|  | *ELOVL2* | rs2236212 | *G/C* | 25 | -0.06 | 0.793 | 18 | 0.02 | 0.922 |
|  | *ELOVL2* | rs3798713 | *G/C* | 25 | -0.06 | 0.793 | 18 | 0.02 | 0.922 |
|  | *ELOVL2* | rs953413 | *A/G* | 21 | -0.15 | 0.543 | 16 | 0.10 | 0.613 |
| **C22:5n-6 (DPAn6)** | |  |  |  |  |  |  |  |  |
|  | *ELOVL2* | rs2236212 | *G/C* | 25 | 0.03 | 0.903 | 19 | 0.02 | 0.928 |
|  | *ELOVL2* | rs3798713 | *G/C* | 25 | 0.10 | 0.637 | 19 | 0.02 | 0.928 |
|  | *ELOVL2* | rs953413 | *A/G* | 21 | -0.09 | 0.679 | 17 | -0.09 | 0.645 |
| **C20:5n3 (EPA)** |  |  |  |  |  |  |  |  |  |
|  | *ELOVL2* | rs2236212 | *G/C* | 25 | -0.15 | 0.479 | 18 | -0.16 | 0.598 |
|  | *ELOVL2* | rs3798713 | *G/C* | 25 | -0.17 | 0.427 | 18 | -0.16 | 0.598 |
|  | *ELOVL2* | rs953413 | *A/G* | 21 | -0.12 | 0.602 | 16 | -0.10 | 0.743 |
| **C22:5n-3 (DPAn3)** | |  |  |  |  |  |  |  |  |
|  | *ELOVL2* | rs2236212 | *G/C* | 25 | -0.32 | 0.114 | 19 | 0.44 | 0.072 |
|  | *ELOVL2* | rs3798713 | *G/C* | 25 | -0.34 | 0.104 | 19 | 0.44 | 0.072 |
|  | *ELOVL2* | rs953413 | *A/G* | 21 | 0.21 | 0.375 | 17 | -0.09 | 0.758 |
| **C22:6n-3 (DHA)** | | | | | | | | | |
|  | *ELOVL2* | rs2236212 | *G/C* | 25 | -0.32 | 0.109 | 19 | -0.02 | 0.943 |
|  | *ELOVL2* | rs3798713 | *G/C* | 25 | -0.29 | 0.176 | 19 | -0.02 | 0.943 |
|  | *ELOVL2* | rs953413 | *A/G* | 21 | 0.04 | 0.869 | 17 | -0.06 | 0.768 |
| **Fatty acids involved in *ELOVL5* indexes** | | |  |  |  |  |  |  |  |
| **C18:2n6 (LA)** |  |  |  |  |  |  |  |  |  |
|  | *ELOVL5* | rs2397142 | *C/G* | 27 | 0.01 | 0.965 | 19 | 0.04 | 0.898 |
|  | *ELOVL5* | rs9395855 | *T/G* | 19 | 0.05 | 0.858 | 17 | -0.31 | 0.232 |
| **C18:3n6 (GLA)** |  |  |  |  |  |  |  |  |  |
|  | *ELOVL5* | rs2397142 | *C/G* | 26 | -0.04 | 0.867 | 19 | -0.18 | 0.518 |
|  | *ELOVL5* | rs9395855 | *T/G* | 18 | 0.02 | 0.949 | 17 | 0.41 | 0.136 |
| **C20:3n-6 (DGLA)** | |  |  |  |  |  |  |  |  |
|  | *ELOVL5* | rs2397142 | *C/G* | 27 | -0.39 | 0.081 | 19 | 0.03 | 0.931 |
|  | *ELOVL5* | rs9395855 | *T/G* | 19 | -0.05 | 0.847 | 17 | 0.09 | 0.766 |
| **C20:4n-6 (AA)** |  |  |  |  |  |  |  |  |  |
|  | *ELOVL5* | rs2397142 | *C/G* | 27 | -0.11 | 0.641 | 19 | -0.30 | 0.241 |
|  | *ELOVL5* | rs9395855 | *T/G* | 19 | 0.44 | 0.101 | 17 | 0.24 | 0.366 |
| **C22:4n-6 (AdA)** | | | | | | | | | |
|  | *ELOVL5* | rs2397142 | *C/G* | 27 | -0.13 | 0.583 | 18 | -0.14 | 0.523 |
|  | *ELOVL5* | rs9395855 | *T/G* | 19 | 0.28 | 0.294 | 16 | 0.04 | 0.845 |
| **C18:3n-3 (ALA)** | | | | | | | | | |
|  | *ELOVL5* | rs2397142 | *C/G* | 27 | 0.15 | 0.546 | 19 | -0.07 | 0.815 |
|  | *ELOVL5* | rs9395855 | *T/G* | 19 | -0.18 | 0.515 | 17 | 0.17 | 0.569 |
| **C20:5n3 (EPA)** |  |  |  |  |  |  |  |  |  |
|  | *ELOVL5* | rs2397142 | *C/G* | 27 | 0.15 | 0.486 | 18 | 0.29 | 0.298 |
|  | *ELOVL5* | rs9395855 | *T/G* | 19 | -0.05 | 0.841 | 16 | -0.36 | 0.187 |
| Associations between SNPs and fatty acids were analyzed using linear regression. SNPs were coded according to minor allele count and analyzed as a numeric variable. "β"= beta per minor allele standardized per the major allele. All associations were adjusted for potential confounders such as age, education, smoking and energy intake. P-values <0.05 are highlighted in bold and significant associations that persisted after Bonferroni correction are additionally denoted by stars (*p<0.004). LA: Linoleic Acid; GLA: γ-Linolenic Acid; DGLA: Dihomo-γ-Linolenic Acid; AA: Arachidonic Acid; AdA: Adrenic Acid; DPAn6: Docosapentaenoic acid n6; ALA: α-linolenic Acid; EPA: Eicosapentaenoic acid; DPAn3: Docosapentaenoic acid n3; DHA: Docosahexaenoic acid. | | | | | | | | | |
|  |  |  |  |  |  |  |  |  |  |
|  |  |  |  |  |  |  |  |  |  |
|  |  |  |  |  |  |  |  |  |  |
|  |  |  |  |  |  |  |  |  |  |
|  |  |  |  |  |  |  |  |  |  |
|  |  |  |  |  |  |  |  |  |  |
