# Supplementary material for: Association of maternal weight with FADS and ELOVL genetic variants and fatty acid levels- The PREOBE follow-up
Source: PLoS One. 2017 Jun 9;12(6):e0179135. doi: 10.1371/journal.pone.0179135 (PMC5466308; doi:10.1371/journal.pone.0179135)
Supplement: S4 Table — (DOCX) [file pone.0179135.s005.docx]

S4 Table. PUFA levels in plasma according to maternal *FADS* and *ELOVL* SNPs.

a) *FADS1* indexes

|  | ***Gene*** | **SNP *Major/minor allele*** | | **NORMAL-WEIGHT** | | | | | **OVERWEIGHT/OBESITY** | | | | | **P (*MM*)** | **P (*Mm+mm*)** |
| --- | --- | --- | --- | --- | --- | --- | --- | --- | --- | --- | --- | --- | --- | --- | --- |
|  |  |  |  | ***MM*** | | ***Mm+mm*** | | **P** | ***MM*** | | ***Mm+mm*** | | **P** |  |  |
|  |  |  |  | **N** | **Mean (SD)** | **N** | **Mean (SD)** |  | **N** | **Mean (SD)** | **N** | **Mean (SD)** |  |  |  |
| ***FADS1* indexes** | | | | | | | | | | | | | | | |
| **AA:LA** | |  |  |  |  |  |  |  |  |  |  |  |  |  |  |
|  | *FADS1* | rs174537 | *G/T* | 15 | 0.45 (0.08) | 23 | 0.39 (0.09) | **0.038** | 12 | 0.46 (0.07) | 21 | 0.43 (0.08) | 0.390 | 0.811 | 0.085 |
|  | *FADS1* | rs174545 | *C/G* | 22 | 0.46 (0.09) | 27 | 0.38 (0.08) | **0.003*** | 13 | 0.47 (0.07) | 22 | 0.42 (0.08) | 0.071 | 0.877 | 0.171 |
|  | *FADS1* | rs174546 | *C/T* | 22 | 0.46 (0.09) | 27 | 0.38 (0.08) | **0.003*** | 13 | 0.47 (0.07) | 25 | 0.42 (0.08) | 0.098 | 0.877 | 0.109 |
|  | *FADS1* | rs174548 | *C/G* | 24 | 0.45 (0.08) | 25 | 0.39 (0.09) | **0.031** | 13 | 0.45 (0.08) | 25 | 0.43 (0.08) | 0.553 | 0.969 | 0.115 |
|  | *FADS1* | rs174553 | *A/G* | 22 | 0.46 (0.09) | 27 | 0.38 (0.08) | **0.003*** | 13 | 0.47 (0.07) | 25 | 0.42 (0.08) | 0.098 | 0.877 | 0.109 |
|  | *FADS1* | rs174561 | *T/C* | 15 | 0.42 (0.07) | 14 | 0.41 (0.09) | 0.832 | 20 | 0.45 (0.11) | 17 | 0.44 (0.11) | 0.939 | 0.332 | 0.336 |
|  | *FADS1* | rs174547 | *T/C* | 15 | 0.45 (0.08) | 23 | 0.39 (0.09) | **0.040** | 12 | 0.46 (0.07) | 20 | 0.43 (0.08) | 0.427 | 0.950 | 0.124 |
| **AA:DGLA** | |  |  |  |  |  |  |  |  |  |  |  |  |  |  |
|  | *FADS1* | rs174537 | *G/T* | 15 | 3.14 (0.70) | 23 | 2.20 (0.60) | **<0.001*** | 12 | 3.57 (1.73) | 21 | 2.55 (0.80) | **0.032** | 0.414 | 0.107 |
|  | *FADS1* | rs174545 | *C/G* | 22 | 3.12 (0.65) | 27 | 2.25 (0.57) | **<0.001*** | 13 | 3.48 (1.67) | 22 | 2.51 (0.78) | **0.025** | 0.362 | 0.183 |
|  | *FADS1* | rs174546 | *C/T* | 22 | 3.12 (0.65) | 27 | 2.25 (0.57) | **<0.001*** | 13 | 3.48 (1.67) | 25 | 2.54 (0.74) | **0.021** | 0.362 | 0.120 |
|  | *FADS1* | rs174548 | *C/G* | 24 | 2.97 (0.65) | 25 | 2.33 (0.70) | **0.002*** | 13 | 3.42 (1.69) | 25 | 2.57 (0.75) | **0.037** | 0.244 | 0.246 |
|  | *FADS1* | rs174553 | *A/G* | 22 | 3.12 (0.65) | 27 | 2.25 (0.57) | **<0.001*** | 13 | 3.48 (1.67) | 25 | 2.54 (0.74) | **0.021** | 0.362 | 0.120 |
|  | *FADS1* | rs174561 | *T/C* | 15 | 2.78 (0.59) | 14 | 2.71 (0.78) | 0.771 | 20 | 2.65 (0.67) | 17 | 2.61 (0.60) | 0.843 | 0.550 | 0.691 |
|  | *FADS1* | rs174547 | *T/C* | 15 | 3.20 (0.67) | 23 | 2.27 (0.55) | **<0.001*** | 12 | 3.54 (1.73) | 20 | 2.56 (0.82) | **0.037** | 0.291 | 0.194 |
| **EPA:ALA** | |  |  |  |  |  |  |  |  |  |  |  |  |  |  |
|  | *FADS1* | rs174537 | *G/T* | 15 | 2.63 (1.29) | 20 | 2.26 (1.03) | 0.351 | 10 | 2.33 (1.17) | 21 | 2.40 (1.43) | 0.904 | 0.568 | 0.721 |
|  | *FADS1* | rs174545 | *C/G* | 21 | 2.74 (1.19) | 23 | 2.26 (0.96) | 0.147 | 11 | 2.31 (1.11) | 22 | 2.33 (1.41) | 0.971 | 0.338 | 0.834 |
|  | *FADS1* | rs174546 | *C/T* | 21 | 2.74 (1.19) | 23 | 2.26 (0.96) | 0.147 | 11 | 2.31 (1.11) | 25 | 2.30 (1.34) | 0.967 | 0.338 | 0.910 |
|  | *FADS1* | rs174548 | *C/G* | 23 | 2.58 (1.06) | 21 | 2.39 (1.14) | 0.571 | 11 | 2.27 (1.09) | 25 | 2.31 (1.35) | 0.927 | 0.442 | 0.848 |
|  | *FADS1* | rs174553 | *A/G* | 21 | 2.74 (1.19) | 23 | 2.26 (0.96) | 0.147 | 11 | 2.31 (1.11) | 25 | 2.30 (1.34) | 0.967 | 0.338 | 0.910 |
|  | *FADS1* | rs174561 | *T/C* | 14 | 2.77 (1.19) | 12 | 2.24 (0.67) | 0.185 | 19 | 2.36 (0.97) | 17 | 2.30 (1.25) | 0.879 | 0.285 | 0.873 |
|  | *FADS1* | rs174547 | *T/C* | 15 | 2.74 (1.32) | 21 | 2.26 (1.00) | 0.220 | 10 | 2.33 (1.17) | 20 | 2.40 (1.47) | 0.898 | 0.437 | 0.713 |
| P-value derived from global ANOVA and significance level (p<0.004) was adjusted for multiple testing by Bonferroni correction. Data are means of FAs expressed as percentages of the total phospholipid profile (standard error). P-values <0.05 are highlighted in bold and significant associations that persisted after Bonferroni correction are additionally denoted by stars or daggers (p<0.004). *Indicates significant differences within each group of weight and † Indicates significant differences between groups of weight. Major allele: M; minor allele: m; LA: Linoleic Acid; DGLA: Dihomo-γ-Linolenic Acid; AA: Arachidonic Acid; ALA: α-linolenic Acid; EPA: Eicosapentaenoic acid. | | | | | | | | | | | | | | | |

b) *FADS2* indexes

|  | ***Gene*** | **SNP *Major/minor allele*** | | **NORMAL-WEIGHT** | | | | | **OVERWEIGHT/OBESITY** | | | | | **P (*MM*)** | **P (*Mm+mm*)** |
| --- | --- | --- | --- | --- | --- | --- | --- | --- | --- | --- | --- | --- | --- | --- | --- |
|  |  |  |  | ***MM*** | | ***Mm+mm*** | | **P** | ***MM*** | | ***Mm+mm*** | | **P** |  |  |
|  |  |  |  | **N** | **Mean (SD)** | **N** | **Mean (SD)** |  | **N** | **Mean (SD)** | **N** | **Mean (SD)** |  |  |  |
| ***FADS2* indexes** | | | | | | | | | | | | | | | |
| **GLA:LA** | |  |  |  |  |  |  |  |  |  |  |  |  |  |  |
|  | *FADS2* | rs1535 | *A/G* | 19 | 0.002 (0.001) | 23 | 0.003 (0.001) | 0.307 | 12 | 0.002 (0.001) | 23 | 0.002 (0.001) | 0.492 | 0.416 | 0.359 |
|  | *FADS2* | rs174575 | *C/G* | 23 | 0.002 (0.001) | 14 | 0.003 (0.001) | 0.266 | 20 | 0.002 (0.001) | 14 | 0.003 (0.001) | 0.260 | 0.870 | 0.746 |
|  | *FADS2* | rs174583 | *C/T* | 18 | 0.002 (0.001) | 25 | 0.002 (0.001) | 0.562 | 12 | 0.002 (0.001) | 25 | 0.002 (0.001) | 0.541 | 0.496 | 0.604 |
|  | *FADS2* | rs99780 | *C/T* | 15 | 0.002 (0.001) | 24 | 0.002 (0.001) | 0.625 | 11 | 0.002 (0.001) | 22 | 0.002 (0.001) | 0.973 | 0.982 | 0.601 |
|  | *FADS2* | rs174602 | *T/C* | 18 | 0.002 (0.001) | 13 | 0.003 (0.001) | 0.879 | 23 | 0.002 (0.001) | 13 | 0.003 (0.001) | 0.465 | 0.590 | 0.932 |
| **DGLA:LA** | |  |  |  |  |  |  |  |  |  |  |  |  |  |  |
|  | *FADS2* | rs1535 | *A/G* | 20 | 0.15 (0.04) | 25 | 0.18 (0.05) | **0.011** | 13 | 0.15 (0.04) | 23 | 0.18 (0.05) | 0.115 | 0.877 | 0.798 |
|  | *FADS2* | rs174575 | *C/G* | 24 | 0.15 (0.04) | 16 | 0.19 (0.06) | **0.017** | 21 | 0.17 (0.06) | 14 | 0.17 (0.05) | 0.943 | 0.276 | 0.315 |
|  | *FADS2* | rs174583 | *C/T* | 19 | 0.15 (0.04) | 27 | 0.18 (0.05) | **0.036** | 13 | 0.15 (0.04) | 25 | 0.18 (0.05) | 0.130 | 0.967 | 0.907 |
|  | *FADS2* | rs99780 | *C/T* | 15 | 0.15 (0.04) | 26 | 0.18 (0.05) | **0.037** | 12 | 0.15 (0.05) | 22 | 0.18 (0.05) | 0.076 | 0.953 | 0.961 |
|  | *FADS2* | rs174602 | *T/C* | 19 | 0.16 (0.04) | 13 | 0.15 (0.05) | 0.678 | 23 | 0.17 (0.05) | 14 | 0.18 (0.03) | 0.811 | 0.323 | 0.124 |
| **AA:LA** | |  |  |  |  |  |  |  |  |  |  |  |  |  |  |
|  | *FADS2* | rs1535 | *A/G* | 20 | 0.45 (0.08) | 25 | 0.39 (0.09) | **0.025** | 13 | 0.47 (0.07) | 23 | 0.42 (0.08) | 0.128 | 0.504 | 0.170 |
|  | *FADS2* | rs174575 | *C/G* | 24 | 0.43 (0.09) | 16 | 0.40 (0.08) | 0.301 | 21 | 0.45 (0.07) | 14 | 0.43 (0.10) | 0.521 | 0.368 | 0.320 |
|  | *FADS2* | rs174583 | *C/T* | 19 | 0.44 (0.08) | 27 | 0.38 (0.08) | **0.019** | 13 | 0.47 (0.07) | 25 | 0.42 (0.08) | 0.098 | 0.447 | 0.109 |
|  | *FADS2* | rs99780 | *C/T* | 15 | 0.44 (0.09) | 26 | 0.40 (0.09) | 0.152 | 12 | 0.46 (0.07) | 22 | 0.43 (0.09) | 0.435 | 0.549 | 0.144 |
|  | *FADS2* | rs174602 | *T/C* | 19 | 0.41 (0.07) | 13 | 0.42 (0.09) | 0.730 | 23 | 0.46 (0.11) | 14 | 0.42 (0.11) | 0.351 | 0.111 | 0.928 |
| **DPAn6:AA** | |  |  |  |  |  |  |  |  |  |  |  |  |  |  |
|  | *FADS2* | rs1535 | *A/G* | 20 | 0.05 (0.01) | 25 | 0.05 (0.01) | 0.178 | 13 | 0.05 (0.02) | 23 | 0.05 (0.02) | 0.428 | 0.943 | 0.765 |
|  | *FADS2* | rs174575 | *C/G* | 24 | 0.05 (0.01) | 16 | 0.05 (0.02) | 0.999 | 21 | 0.05 (0.01) | 14 | 0.05 (0.02) | 0.627 | 0.690 | 0.863 |
|  | *FADS2* | rs174583 | *C/T* | 19 | 0.05 (0.01) | 27 | 0.05 (0.01) | 0.371 | 13 | 0.05 (0.02) | 25 | 0.05 (0.02) | 0.470 | 0.787 | 0.813 |
|  | *FADS2* | rs99780 | *C/T* | 15 | 0.05 (0.01) | 26 | 0.05 (0.01) | 0.364 | 12 | 0.05 (0.02) | 22 | 0.05 (0.02) | 0.359 | 0.762 | 0.987 |
|  | *FADS2* | rs174602 | *T/C* | 19 | 0.05 (0.01) | 13 | 0.05 (0.02) | 0.979 | 23 | 0.05 (0.01) | 14 | 0.05 (0.01) | 0.731 | 0.621 | 0.533 |
| **DPAn6:AdA** | |  |  |  |  |  |  |  |  |  |  |  |  |  |  |
|  | *FADS2* | rs1535 | *A/G* | 20 | 1.27 (0.17) | 24 | 1.25 (0.29) | 0.839 | 13 | 1.22 (0.24) | 22 | 1.25 (0.22) | 0.721 | 0.560 | 0.980 |
|  | *FADS2* | rs174575 | *C/G* | 24 | 1.28 (0.24) | 15 | 1.21 (0.27) | 0.354 | 21 | 1.25 (0.22) | 13 | 1.26 (0.23) | 0.880 | 0.276 | 0.775 |
|  | *FADS2* | rs174583 | *C/T* | 19 | 1.28 (0.17) | 26 | 1.24 (0.28) | 0.566 | 13 | 1.22 (0.24) | 24 | 1.25 (0.23) | 0.766 | 0.455 | 0.868 |
|  | *FADS2* | rs99780 | *C/T* | 15 | 1.29 (0.16) | 25 | 1.23 (0.27) | 0.441 | 12 | 1.22 (0.25) | 21 | 1.25 (0.22) | 0.759 | 0.371 | 0.847 |
|  | *FADS2* | rs174602 | *T/C* | 19 | 1.28 (0.22) | 12 | 1.27 (0.27) | 0.939 | 22 | 1.20 (0.23) | 14 | 0.20 (0.18) | 0.959 | 0.285 | 0.475 |
| **EPA:ALA** | |  |  |  |  |  |  |  |  |  |  |  |  |  |  |
|  | *FADS2* | rs1535 | *A/G* | 19 | 2.74 (1.24) | 22 | 2.24 (0.98) | 0.162 | 11 | 2.31 (1.11) | 23 | 2.36 (1.38) | 0.923 | 0.361 | 0.738 |
|  | *FADS2* | rs174575 | *C/G* | 23 | 2.43 (1.19) | 13 | 2.18 (0.78) | 0.509 | 19 | 2.12 (0.91) | 14 | 2.65 (1.69) | 0.256 | 0.365 | 0.369 |
|  | *FADS2* | rs174583 | *C/T* | 19 | 2.74 (1.24) | 23 | 2.26 (0.96) | 0.167 | 11 | 2.31 (1.11) | 25 | 2.30 (1.34) | 0.967 | 0.361 | 0.910 |
|  | *FADS2* | rs99780 | *C/T* | 15 | 2.40 (1.07) | 23 | 2.49 (1.21) | 0.808 | 10 | 2.24 (1.14) | 22 | 2.44 (1.40) | 0.688 | 0.726 | 0.903 |
|  | *FADS2* | rs174602 | *T/C* | 18 | 2.39 (1.07) | 11 | 2.52 (0.87) | 0.729 | 23 | 2.29 (1.03) | 13 | 2.42 (1.24) | 0.738 | 0.754 | 0.811 |
| **DHA:EPA** | |  |  |  |  |  |  |  |  |  |  |  |  |  |  |
|  | *FADS2* | rs1535 | *A/G* | 20 | 15.02 (5.64) | 25 | 14.66 (5.92) | 0.840 | 13 | 19.69 (6.63) | 23 | 20.89 (7.87) | 0.655 | **0.045** | **0.004†** |
|  | *FADS2* | rs174575 | *C/G* | 24 | 16.41 (5.99) | 16 | 13.41 (5.21) | 0.119 | 21 | 20.99 (6.36) | 14 | 20.27 (8.32) | 0.778 | **0.018** | **0.012** |
|  | *FADS2* | rs174583 | *C/T* | 19 | 15.02 (5.64) | 27 | 14.48 (5.71) | 0.753 | 13 | 19.69 (6.63) | 25 | 20.58 (7.89) | 0.737 | **0.045** | **0.003†** |
|  | *FADS2* | rs99780 | *C/T* | 15 | 16.22 (5.52) | 26 | 13.71 (4.76) | 0.138 | 12 | 20.50 (6.29) | 22 | 20.19 (8.14) | 0.911 | 0.078 | **0.002†** |
|  | *FADS2* | rs174602 | *T/C* | 19 | 16.06 (5.17) | 13 | 14.76 (6.12) | 0.531 | 23 | 19.97 (6.82) | 14 | 18.02 (6.37) | 0.393 | **0.046** | 0.199 |
| **DHA:DPAn3** | |  |  |  |  |  |  |  |  |  |  |  |  |  |  |
|  | *FADS2* | rs1535 | *A/G* | 20 | 10.84 (1.96) | 25 | 10.03 (2.40) | 0.232 | 13 | 11.14 (2.26) | 23 | 11.58 (2.26) | 0.577 | 0.687 | **0.027** |
|  | *FADS2* | rs174575 | *C/G* | 24 | 10.58 ( 2.21) | 16 | 10.15 (2.39) | 0.574 | 21 | 11.23 (2.29) | 14 | 12.20 (2.28) | 0.230 | 0.334 | **0.026** |
|  | *FADS2* | rs174583 | *C/T* | 19 | 10.76 (1.98) | 27 | 10.13 (2.33) | 0.350 | 13 | 11.14 (2.26) | 25 | 11.53 (2.50) | 0.642 | 0.613 | **0.044** |
|  | *FADS2* | rs99780 | *C/T* | 15 | 11.04 (2.07) | 26 | 9.83 (2.17) | 0.090 | 12 | 11.42 (2.11) | 22 | 11.41 (2.45) | 0.990 | 0.640 | **0.024** |
|  | *FADS2* | rs174602 | *T/C* | 19 | 11.04 (2.08) | 13 | 10.67 (2.43) | 0.641 | 23 | 11.14 (2.75) | 14 | 11.40 (2.50) | 0.768 | 0.902 | 0.443 |
| P-value derived from global ANOVA and significance level (p<0.004) was adjusted for multiple testing by Bonferroni correction. Data are means of FAs expressed as percentages of the total phospholipid profile (standard error). P-values <0.05 are highlighted in bold and significant associations that persisted after Bonferroni correction are additionally denoted by stars or daggers (p<0.004). *Indicates significant differences within each group of weight and † Indicates significant differences between groups of weight. Major allele: M; minor allele: m; LA: Linoleic Acid; GLA: γ-Linolenic Acid; DGLA: Dihomo-γ-Linolenic Acid; AA: Arachidonic Acid; AdA: Adrenic Acid; DPAn6: Docosapentaenoic acid n6; ALA: α-linolenic Acid; EPA: Eicosapentaenoic acid; DPAn3: Docosapentaenoic acid n3; DHA: Docosahexaenoic acid. | | | | | | | | | | | | | | | |

c) *ELOVL* indexes

|  | ***Gene*** | **SNP *Major/minor allele*** | | **NORMAL-WEIGHT** | | | | | **OVERWEIGHT/OBESITY** | | | | | **P (*MM*)** | **P (*Mm+mm*)** |
| --- | --- | --- | --- | --- | --- | --- | --- | --- | --- | --- | --- | --- | --- | --- | --- |
|  |  |  |  | ***MM*** | | ***Mm+mm*** | | **P** | ***MM*** | | ***Mm+mm*** | | **P** |  |  |
|  |  |  |  | **N** | **Mean (SD)** | **N** | **Mean (SD)** |  | **N** | **Mean (SD)** | **N** | **Mean (SD)** |  |  |  |
| ***ELOVL2* indexes** | | |  |  |  |  |  |  |  |  |  |  |  |  |  |
| **DPAn6:AdA** | |  |  |  |  |  |  |  |  |  |  |  |  |  |  |
|  | *ELOVL2* | rs2236212 | *G/C* | 14 | 1.30 (0.29) | 29 | 1.27 (0.25) | 0.732 | 13 | 1.26 (0.18) | 22 | 1.23 (0.26) | 0.726 | 0.654 | 0.562 |
|  | *ELOVL2* | rs3798713 | *G/C* | 11 | 1.30 (0.28) | 31 | 1.24 (0.21) | 0.438 | 12 | 1.26 (0.19) | 25 | 1.23 (0.25) | 0.724 | 0.659 | 0.889 |
|  | *ELOVL2* | rs953413 | *A/G* | 10 | 1.19 (0.28) | 26 | 1.29 (0.23) | 0.261 | 7 | 1.30 (0.28) | 20 | 1.23 (0.21) | 0.527 | 0.442 | 0.386 |
| **DPAn3:EPA** | |  |  |  |  |  |  |  |  |  |  |  |  |  |  |
|  | *ELOVL2* | rs2236212 | *G/C* | 14 | 1.51 (0.51) | 29 | 1.48 (0.67) | 0.876 | 13 | 1.66 (0.71) | 22 | 1.88 (0.71) | 0.388 | 0.530 | **0.047** |
|  | *ELOVL2* | rs3798713 | *G/C* | 11 | 1.48 (0.52) | 31 | 1.44 (0.67) | 0.863 | 12 | 1.67 (0.74) | 25 | 1.88 (0.70) | 0.410 | 0.476 | **0.021** |
|  | *ELOVL2* | rs953413 | *A/G* | 10 | 1.64 (0.73) | 26 | 1.46 (0.62) | 0.479 | 7 | 2.11 (1.01) | 20 | 1.73 (0.63) | 0.244 | 0.278 | 0.166 |
| **DHA:EPA** | |  |  |  |  |  |  |  |  |  |  |  |  |  |  |
|  | *ELOVL2* | rs2236212 | *G/C* | 14 | 16.39 (6.29) | 28 | 14.45 (5.43) | 0.307 | 13 | 20.58 (8.33) | 22 | 20.05 (7.02) | 0.841 | 0.151 | **0.003†** |
|  | *ELOVL2* | rs3798713 | *G/C* | 11 | 15.48 (5.10) | 30 | 13.96 (5.33) | 0.418 | 12 | 20.76 (8.67) | 24 | 20.13 (7.07) | 0.819 | 0.094 | **<0.001†** |
|  | *ELOVL2* | rs953413 | *A/G* | 10 | 15.33 (5.93) | 25 | 14.78 (5.23) | 0.789 | 7 | 21.14 (7.60) | 20 | 20.88 (7.57) | 0.939 | 0.096 | **0.003†** |
| **DHA:DPAn3** | |  |  |  |  |  |  |  |  |  |  |  |  |  |  |
|  | *ELOVL2* | rs2236212 | *G/C* | 14 | 10.87 (2.20) | 29 | 10.14 (2.32) | 0.337 | 13 | 12.57 (1.53) | 22 | 10.77 (2.67) | **0.032** | **0.029** | 0.375 |
|  | *ELOVL2* | rs3798713 | *G/C* | 11 | 10.68 (2.24) | 31 | 10.10 (2.20) | 0.460 | 12 | 12.64 (1.58) | 25 | 10.78 (2.55) | **0.028** | **0.024** | 0.291 |
|  | *ELOVL2* | rs953413 | *A/G* | 10 | 9.83 (2.22) | 26 | 10.38 (1.99) | 0.478 | 7 | 10.68 (2.80) | 20 | 12.04 (2.09) | 0.183 | 0.493 | **0.008** |
| ***ELOVL5* indexes** | | |  |  |  |  |  |  |  |  |  |  |  |  |  |
| **DGLA:LA** | |  |  |  |  |  |  |  |  |  |  |  |  |  |  |
|  | *ELOVL5* | rs2397142 | *C/G* | 24 | 0.17 (0.05) | 21 | 0.16 (0.04) | 0.380 | 19 | 0.17 (0.06) | 17 | 0.17 (0.04) | 0.786 | 0.730 | 0.424 |
|  | *ELOVL5* | rs9395855 | *T/G* | 6 | 0.19 (0.04) | 25 | 0.16 (0.04) | 0.246 | 8 | 0.17 (0.04) | 21 | 0.17 (0.06) | 0.930 | 0.579 | 0.593 |
| **DGLA:GLA** | |  |  |  |  |  |  |  |  |  |  |  |  |  |  |
|  | *ELOVL5* | rs2397142 | *C/G* | 22 | 86.68 (33.02) | 21 | 75.75 (33.15) | 0.436 | 19 | 76.97 (35.87) | 15 | 75.73 (18.30) | 0.904 | 0.537 | 0.999 |
|  | *ELOVL5* | rs9395855 | *T/G* | 6 | 87.14 (52.95) | 23 | 73.81 (30.40) | 0.422 | 7 | 75.33 (21.41) | 20 | 77.97 (34.17) | 0.850 | 0.597 | 0.675 |
| **AA:LA** | |  |  |  |  |  |  |  |  |  |  |  |  |  |  |
|  | *ELOVL5* | rs2397142 | *C/G* | 24 | 0.42 (0.08) | 21 | 0.39 (0.09) | 0.207 | 19 | 0.45 (0.08) | 17 | 0.42 (0.08) | 0.285 | 0.252 | 0.235 |
|  | *ELOVL5* | rs9395855 | *T/G* | 6 | 0.39 (0.14) | 25 | 0.42 (0.09) | 0.504 | 8 | 0.41 (0.07) | 21 | 0.46 (0.08) | 0.174 | 0.760 | 0.188 |
| **AdA:AA** | |  |  |  |  |  |  |  |  |  |  |  |  |  |  |
|  | *ELOVL5* | rs2397142 | *C/G* | 24 | 0.04 (0.01) | 21 | 0.04 (0.01) | 0.934 | 18 | 0.04 (0.01) | 17 | 0.04 (0.01) | 0.469 | 0.367 | 0.937 |
|  | *ELOVL5* | rs9395855 | *T/G* | 6 | 0.04 (0.01) | 25 | 0.04 (0.01) | 0.653 | 8 | 0.04 (0.01) | 20 | 0.04 (0.01) | 0.197 | 0.917 | 0.252 |
| **EPA:ALA** | |  |  |  |  |  |  |  |  |  |  |  |  |  |  |
|  | *ELOVL5* | rs2397142 | *C/G* | 23 | 2.50 (1.13) | 19 | 2.44 (1.12) | 0.858 | 18 | 2.04 (0.74) | 16 | 2.61 (1.69) | 0.202 | 0.142 | 0.719 |
|  | *ELOVL5* | rs9395855 | *T/G* | 5 | 2.16 (0.50) | 25 | 2.41 (1.27) | 0.673 | 8 | 2.97 (2.13) | 19 | 2.28 (0.91) | 0.241 | 0.425 | 0.717 |
| P-value derived from global ANOVA and significance level (p<0.004) was adjusted for multiple testing by Bonferroni correction. Data are means of FAs expressed as percentages of the total phospholipid profile (standard error). P-values <0.05 are highlighted in bold and significant associations that persisted after Bonferroni correction are additionally denoted by stars or daggers (p<0.004). *Indicates significant differences within each group of weight and † Indicates significant differences between groups of weight. Major allele: M; minor allele: m; LA: Linoleic Acid; GLA: γ-Linolenic Acid; DGLA: Dihomo-γ-Linolenic Acid; AA: Arachidonic Acid; AdA: Adrenic Acid; DPAn6: Docosapentaenoic acid n6; ALA: α-linolenic Acid; EPA: Eicosapentaenoic acid; DPAn3: Docosapentaenoic acid n3; DHA: Docosahexaenoic acid. | | | | | | | | | | | | | | | |

d) Fatty acids involved in *FADS1* indexes

|  | ***Gene*** | **SNP *Major/minor allele*** | | **NORMAL-WEIGHT** | | | | | **OVERWEIGHT/OBESITY** | | | | | **P (*MM*)** | **P (*Mm+mm*)** |
| --- | --- | --- | --- | --- | --- | --- | --- | --- | --- | --- | --- | --- | --- | --- | --- |
|  |  |  |  | ***MM*** | | ***Mm+mm*** | | **P** | ***MM*** | | ***Mm+mm*** | | **P** |  |  |
|  |  |  |  | **N** | **Mean (SD)** | **N** | **Mean (SD)** |  | **N** | **Mean (SD)** | **N** | **Mean (SD)** |  |  |  |
| **Fatty acids involved in *FADS1* indexes** | | | | | | | | | | | | | | | |
| **C18:2n-6 (LA)** | | | | | | | | | | | | | | | |
|  | *FADS1* | rs174537 | *G/T* | 15 | 23.77 (2.02) | 23 | 23.56 (2.39) | 0.782 | 12 | 23.35 (2.12) | 21 | 23.50 (2.42) | 0.855 | 0.603 | 0.937 |
|  | *FADS1* | rs174545 | *C/G* | 22 | 23.11 (2.19) | 27 | 23.58 (2.21) | 0.460 | 13 | 23.13 (2.17) | 22 | 23.93 (2.50) | 0.345 | 0.975 | 0.602 |
|  | *FADS1* | rs174546 | *C/T* | 22 | 23.11 (2.19) | 27 | 23.58 (2.21) | 0.460 | 13 | 23.13 (2.17) | 25 | 23.84 (2.41) | 0.380 | 0.975 | 0.684 |
|  | *FADS1* | rs174548 | *C/G* | 24 | 23.26 (2.17) | 25 | 23.47 (2.25) | 0.736 | 13 | 23.60 (2.30) | 25 | 23.60 (2.39) | 0.998 | 0.657 | 0.848 |
|  | *FADS1* | rs174553 | *A/G* | 22 | 23.11 (2.19) | 27 | 23.58 (2.21) | 0.460 | 13 | 23.13 (2.17) | 25 | 23.84 (2.41) | 0.380 | 0.975 | 0.684 |
|  | *FADS1* | rs174561 | *T/C* | 15 | 23.92 (1.79) | 14 | 23.11 (2.47) | 0.321 | 20 | 23.43 (2.52) | 17 | 23.80 (2.97) | 0.683 | 0.524 | 0.497 |
|  | *FADS1* | rs174547 | *T/C* | 15 | 23.39 (2.22) | 23 | 23.66 (2.32) | 0.730 | 12 | 23.35 (2.12) | 20 | 23.33 (2.35) | 0.989 | 0.955 | 0.652 |
| **C20:3n-6 (DGLA)** | | |  |  |  |  |  |  |  |  |  |  |  |  |  |
|  | *FADS1* | rs174537 | *G/T* | 15 | 3.51 (0.81) | 23 | 4.27 (0.85) | **0.010** | 12 | 3.31 (0.80) | 21 | 4.18 (0.96) | **0.012** | 0.529 | 0.754 |
|  | *FADS1* | rs174545 | *C/G* | 22 | 3.50 (0.79) | 27 | 4.14 (0.86) | **0.009** | 13 | 3.39 (0.81) | 22 | 4.15 (0.94) | **0.021** | 0.691 | 0.976 |
|  | *FADS1* | rs174546 | *C/T* | 22 | 3.50 (0.79) | 27 | 4.14 (0.86) | **0.009** | 13 | 3.39 (0.81) | 25 | 4.11 (0.90) | **0.021** | 0.691 | 0.893 |
|  | *FADS1* | rs174548 | *C/G* | 24 | 3.59 (0.79) | 25 | 4.10 (0.93) | **0.041** | 13 | 3.38 (0.81) | 25 | 4.11 (0.90) | **0.019** | 0.431 | 0.969 |
|  | *FADS1* | rs174553 | *A/G* | 22 | 3.50 (0.79) | 27 | 4.14 (0.86) | **0.009** | 13 | 3.39 (0.81) | 25 | 4.11 (0.90) | **0.021** | 0.691 | 0.893 |
|  | *FADS1* | rs174561 | *T/C* | 15 | 3.70 (0.90) | 14 | 3.63 (0.83) | 0.828 | 20 | 3.99 (0.64) | 17 | 4.09 (0.76) | 0.662 | 0.270 | 0.115 |
|  | *FADS1* | rs174547 | *T/C* | 15 | 3.61 (0.78) | 23 | 4.17 (0.76) | **0.036** | 12 | 3.31 (0.80) | 20 | 4.17 (0.98) | **0.016** | 0.335 | 0.980 |
| **C20:4n-6 (AA)** | | | | | | | | | | | | | | | |
|  | *FADS1* | rs174537 | *G/T* | 15 | 10.55 (1.33) | 23 | 9.02 (1.54) | **0.003*** | 12 | 10.53 (0.89) | 21 | 10.04 (1.36) | 0.273 | 0.973 | **0.026** |
|  | *FADS1* | rs174545 | *C/G* | 22 | 10.50 (1.39) | 27 | 8.96 (1.49) | **0.001*** | 13 | 10.64 (0.93) | 22 | 9.83 (1.31) | 0.061 | 0.759 | **0.037** |
|  | *FADS1* | rs174546 | *C/T* | 22 | 10.50 (1.39) | 27 | 8.96 (1.49) | **0.001*** | 13 | 10.64 (0.93) | 25 | 9.90 (1.31) | 0.080 | 0.759 | **0.019** |
|  | *FADS1* | rs174548 | *C/G* | 24 | 10.28 (1.39) | 25 | 9.05 (1.64) | **0.007** | 13 | 10.40 (1.02) | 25 | 10.03 (1.33) | 0.389 | 0.790 | **0.025** |
|  | *FADS1* | rs174553 | *A/G* | 22 | 10.50 (1.39) | 27 | 8.96 (1.49) | **0.001*** | 13 | 10.64 (0.93) | 25 | 9.90 (1.31) | 0.080 | 0.759 | **0.019** |
|  | *FADS1* | rs174561 | *T/C* | 15 | 9.88 (1.32) | 14 | 9.30 (1.12) | 0.212 | 20 | 10.27 (1.48) | 17 | 10.33 (1.54) | 0.910 | 0.423 | **0.046** |
|  | *FADS1* | rs174547 | *T/C* | 15 | 10.49 (1.33) | 23 | 9.18 (1.47) | **0.009** | 12 | 10.53 (0.89) | 20 | 10.00 (1.38) | 0.243 | 0.917 | 0.067 |
| **C18:3n-3 (ALA)** | | | | | | | | | | | | | | | |
|  | *FADS1* | rs174537 | *G/T* | 15 | 0.12 (0.04) | 23 | 0.16 (0.04) | **0.021** | 12 | 0.11 (0.03) | 21 | 0.11 (0.04) | 0.632 | 0.496 | **<0.001†** |
|  | *FADS1* | rs174545 | *C/G* | 22 | 0.12 (0.04) | 27 | 0.15 (0.04) | **0.018** | 13 | 0.12 (0.03) | 22 | 0.11 (0.04) | 0.532 | 0.536 | **0.001†** |
|  | *FADS1* | rs174546 | *C/T* | 22 | 0.12 (0.04) | 27 | 0.15 (0.04) | **0.018** | 13 | 0.12 (0.03) | 25 | 0.11 (0.04) | 0.580 | 0.536 | **0.001†** |
|  | *FADS1* | rs174548 | *C/G* | 24 | 0.13 (0.04) | 22 | 0.15 (0.04) | 0.146 | 12 | 0.12 (0.03) | 25 | 0.11 (0.04) | 0.454 | 0.371 | **0.001** |
|  | *FADS1* | rs174553 | *A/G* | 22 | 0.12 (0.04) | 27 | 0.15 (0.04) | **0.018** | 13 | 0.12 (0.03) | 25 | 0.11 (0.04) | 0.580 | 0.536 | **0.001†** |
|  | *FADS1* | rs174561 | *T/C* | 15 | 0.13 (0.04) | 14 | 0.13 (0.03) | 0.695 | 20 | 0.12 (0.05) | 17 | 0.11 (0.03) | 0.497 | 0.397 | **0.024** |
|  | *FADS1* | rs174547 | *T/C* | 15 | 0.12 (0.04) | 23 | 0.15 (0.04) | **0.027** | 12 | 0.11 (0.03) | 20 | 0.11 (0.04) | 0.734 | 0.444 | **<0.001†** |
| **C20:5n3 (EPA)** | | | | | | | | | | | | | | | |
|  | *FADS1* | rs174537 | *G/T* | 15 | 0.31 (0.16) | 23 | 0.33 (0.14) | 0.683 | 11 | 0.25 (0.11) | 21 | 0.26 (0.20) | 0.919 | 0.254 | 0.140 |
|  | *FADS1* | rs174545 | *C/G* | 21 | 0.33 (0.14) | 27 | 0.33 (0.13) | 0.922 | 12 | 0.26 (0.10) | 22 | 0.26 (0.20) | 0.999 | 0.126 | 0.112 |
|  | *FADS1* | rs174546 | *C/T* | 21 | 0.33 (0.14) | 27 | 0.33 (0.13) | 0.922 | 12 | 0.26 (0.10) | 25 | 0.25 (0.19) | 0.937 | 0.126 | 0.074 |
|  | *FADS1* | rs174548 | *C/G* | 23 | 0.32 (0.13) | 25 | 0.34 (0.14) | 0.646 | 12 | 0.26 (0.10) | 25 | 0.25 (0.19) | 0.937 | 0.145 | 0.063 |
|  | *FADS1* | rs174553 | *A/G* | 21 | 0.33 (0.14) | 27 | 0.33 (0.13) | 0.922 | 12 | 0.26 (0.10) | 25 | 0.25 (0.19) | 0.937 | 0.126 | 0.074 |
|  | *FADS1* | rs174561 | *T/C* | 15 | 0.33 (0.14) | 13 | 0.31 (0.11) | 0.683 | 20 | 0.26 (0.11) | 17 | 0.23 (0.10) | 0.491 | 0.092 | **0.044** |
|  | *FADS1* | rs174547 | *T/C* | 15 | 0.33 (0.16) | 23 | 0.32 (0.12) | 0.815 | 11 | 0.25 (0.11) | 20 | 0.26 (0.20) | 0.863 | 0.160 | 0.250 |
| P-value derived from global ANOVA and significance level (p<0.004) was adjusted for multiple testing by Bonferroni correction. Data are means of FAs expressed as percentages of the total phospholipid profile (standard error). P-values <0.05 are highlighted in bold and significant associations that persisted after Bonferroni correction are additionally denoted by stars or daggers (p<0.004). *Indicates significant differences within each group of weight and † Indicates significant differences between groups of weight. Major allele: M; minor allele: m; LA: Linoleic Acid; DGLA: Dihomo-γ-Linolenic Acid; AA: Arachidonic Acid; ALA: α-linolenic Acid; EPA: Eicosapentaenoic acid. | | | | | | | | | | | | | | | |

e) Fatty acids involved in *FADS2* indexes

|  | ***Gene*** | **SNP *Major/minor allele*** | | **NORMAL-WEIGHT** | | | | | **OVERWEIGHT/OBESITY** | | | | | **P (*MM*)** | **P (*Mm+mm*)** |
| --- | --- | --- | --- | --- | --- | --- | --- | --- | --- | --- | --- | --- | --- | --- | --- |
|  |  |  |  | ***MM*** | | ***Mm+mm*** | | **P** | ***MM*** | | ***Mm+mm*** | | **P** |  |  |
|  |  |  |  | **N** | **Mean (SD)** | **N** | **Mean (SD)** |  | **N** | **Mean (SD)** | **N** | **Mean (SD)** |  |  |  |
| **Fatty acids involved in *FADS2* indexes** | | | | | | | | | | | | | | | |
| **C18:2n-6 (LA)** | | | | | | | | | | | | | | | |
|  | *FADS2* | rs1535 | *A/G* | 20 | 23.35 (2.15) | 25 | 23.56 (2.30) | 0.751 | 13 | 23.13 (2.17) | 23 | 23.81 (2.52) | 0.425 | 0.781 | 0.728 |
|  | *FADS2* | rs174575 | *C/G* | 24 | 23.82 (2.06) | 16 | 23.06 (2.41) | 0.291 | 21 | 23.39 (2.37) | 14 | 23.61 (2.39) | 0.795 | 0.519 | 0.538 |
|  | *FADS2* | rs174583 | *C/T* | 19 | 23.54 (2.02) | 27 | 23.58 (2.21) | 0.955 | 13 | 23.13 (2.17) | 25 | 23.84 (2.41) | 0.380 | 0.588 | 0.684 |
|  | *FADS2* | rs99780 | *C/T* | 15 | 23.98 (1.98) | 26 | 23.36 (2.35) | 0.396 | 12 | 23.32 (2.15) | 22 | 23.39 (2.43) | 0.932 | 0.412 | 0.970 |
|  | *FADS2* | rs174602 | *T/C* | 19 | 23.89 (2.07) | 13 | 23.27 (2.39) | 0.443 | 23 | 23.38 (3.02) | 14 | 23.95 (2.15) | 0.545 | 0.541 | 0.444 |
| **C18:3n-6 (GLA)** | | | | | | | | | | | | | | | |
|  | *FADS2* | rs1535 | *A/G* | 19 | 0.05 (0.02) | 24 | 0.06 (0.02) | 0.349 | 12 | 0.06 (0.01) | 23 | 0.05 (0.02) | 0.685 | 0.525 | 0.460 |
|  | *FADS2* | rs174575 | *C/G* | 23 | 0.05 (0.02) | 15 | 0.06 (0.03) | 0.493 | 20 | 0.05 (0.02) | 14 | 0.06 (0.02) | 0.164 | 0.645 | 0.942 |
|  | *FADS2* | rs174583 | *C/T* | 18 | 0.05 (0.02) | 26 | 0.06 (0.02) | 0.660 | 12 | 0.06 (0.01) | 25 | 0.05 (0.02) | 0.765 | 0.648 | 0.767 |
|  | *FADS2* | rs99780 | *C/T* | 15 | 0.06 (0.02) | 25 | 0.06 (0.02) | 0.840 | 11 | 0.05 (0.01) | 22 | 0.05 (0.02) | 0.939 | 0.810 | 0.653 |
|  | *FADS2* | rs174602 | *T/C* | 18 | 0.06 (0.02) | 13 | 0.06 (0.02) | 0.883 | 23 | 0.05 (0.02) | 13 | 0.06 (0.02) | 0.229 | 0.307 | 0.723 |
| **C20:3n-6 (DGLA)** | | |  |  |  |  |  |  |  |  |  |  |  |  |  |
|  | *FADS2* | rs1535 | *A/G* | 20 | 3.41 (0.77) | 25 | 4.23 (0.82) | **0.001*** | 13 | 3.39 (0.81) | 23 | 4.15 (0.92) | **0.019** | 0.927 | 0.736 |
|  | *FADS2* | rs174575 | *C/G* | 24 | 3.57 (0.71) | 16 | 4.28 (0.97) | **0.012** | 21 | 3.82 (1.30) | 14 | 3.90 (0.90) | 0.805 | 0.358 | 0.283 |
|  | *FADS2* | rs174583 | *C/T* | 19 | 3.47 (0.75) | 27 | 4.14 (0.86) | **0.009** | 13 | 3.39 (0.81) | 25 | 4.11 (0.90) | **0.021** | 0.762 | 0.893 |
|  | *FADS2* | rs99780 | *C/T* | 15 | 3.51 (0.78) | 26 | 4.16 (0.89) | **0.025** | 12 | 3.35 (0.84) | 22 | 4.14 (0.94) | **0.022** | 0.613 | 0.941 |
|  | *FADS2* | rs174602 | *T/C* | 19 | 3.77 (0.82) | 13 | 3.50 (0.85) | 0.387 | 23 | 3.95 (0.76) | 14 | 4.19 (0.54) | 0.297 | 0.463 | **0.018** |
| **C20:4n-6 (AA)** | | | | | | | | | | | | | | | |
|  | *FADS2* | rs1535 | *A/G* | 20 | 10.31 (1.30) | 25 | 9.07 (1.50) | **0.005** | 13 | 10.64 (0.93) | 23 | 9.92 (1.35) | 0.102 | 0.439 | **0.044** |
|  | *FADS2* | rs174575 | *C/G* | 24 | 10.02 (1.55) | 15 | 9.12 (1.30) | 0.063 | 21 | 10.37 (0.97) | 14 | 10.01 (1.64) | 0.413 | 0.374 | 0.109 |
|  | *FADS2* | rs174583 | *C/T* | 19 | 10.33 (1.34) | 27 | 8.96 (1.49) | **0.003*** | 13 | 10.64 (0.93) | 25 | 9.90 (1.31) | 0.080 | 0.482 | **0.019** |
|  | *FADS2* | rs99780 | *C/T* | 15 | 10.39 (1.48) | 26 | 9.15 (1.51) | **0.015** | 12 | 10.54 (0.91) | 22 | 10.02 (1.40) | 0.254 | 0.757 | **0.046** |
|  | *FADS2* | rs174602 | *T/C* | 19 | 9.73 (1.34) | 13 | 9.66 (1.23) | 0.874 | 23 | 10.48 (1.39) | 14 | 10.00 (1.66) | 0.347 | 0.085 | 0.553 |
| **C22:4n-6 (AdA)** | | | | | | | | | | | | | | | |
|  | *FADS2* | rs1535 | *A/G* | 20 | 0.39 (0.07) | 25 | 0.40 (0.10) | 0.742 | 13 | 0.41 (0.10) | 22 | 0.40 (0.09) | 0.781 | 0.430 | 0.817 |
|  | *FADS2* | rs174575 | *C/G* | 24 | 0.40 (0.07) | 16 | 0.40 (0.12) | 0.946 | 21 | 0.42 (0.10) | 13 | 0.39 (0.09) | 0.459 | 0.575 | 0.543 |
|  | *FADS2* | rs174583 | *C/T* | 19 | 0.39 (0.07) | 27 | 0.39 (0.10) | 0.865 | 13 | 0.41 (0.10) | 24 | 0.40 (0.09) | 0.753 | 0.553 | 0.644 |
|  | *FADS2* | rs99780 | *C/T* | 15 | 0.39 (0.07) | 26 | 0.40 (0.10) | 0.685 | 12 | 0.40 (0.10) | 21 | 0.41 (0.09) | 0.784 | 0.649 | 0.651 |
|  | *FADS2* | rs174602 | *T/C* | 19 | 0.39 (0.13) | 12 | 0.39 (0.09) | 0.962 | 22 | 0.42 (0.09) | 14 | 0.39 (0.08) | 0.318 | 0.467 | 0.926 |
| **C22:5n-6 (DPAn6)** | | |  |  |  |  |  |  |  |  |  |  |  |  |  |
|  | *FADS2* | rs1535 | *A/G* | 20 | 0.50 (0.14) | 25 | 0.50 (0.14) | 0.966 | 13 | 0.51 (0.17) | 23 | 0.52 (0.15) | 0.922 | 0.782 | 0.594 |
|  | *FADS2* | rs174575 | *C/G* | 24 | 0.51 (0.14) | 16 | 0.48 (0.15) | 0.435 | 21 | 0.52 (0.14) | 14 | 0.52 (0.19) | 0.986 | 0.935 | 0.507 |
|  | *FADS2* | rs174583 | *C/T* | 19 | 0.51 (0.13) | 27 | 0.48 (0.14) | 0.534 | 13 | 0.51 (0.17) | 25 | 0.51 (0.15) | 0.990 | 0.943 | 0.471 |
|  | *FADS2* | rs99780 | *C/T* | 15 | 0.51 (0.14) | 26 | 0.49 (0.14) | 0.758 | 12 | 0.50 (0.18) | 22 | 0.53 (0.16) | 0.642 | 0.921 | 0.408 |
|  | *FADS2* | rs174602 | *T/C* | 19 | 0.49 (0.14) | 13 | 0.49 (0.16) | 0.942 | 23 | 0.51 (0.15) | 14 | 0.47 (0.12) | 0.361 | 0.692 | 0.686 |
| **C18:3n-3 (ALA)** | | | | | | | | | | | | | | | |
|  | *FADS2* | rs1535 | *A/G* | 20 | 0.13 (0.04) | 25 | 0.15 (0.04) | **0.025** | 13 | 0.12 (0.03) | 23 | 0.11 (0.04) | 0.448 | 0.500 | **<0.001†** |
|  | *FADS2* | rs174575 | *C/G* | 24 | 0.13 (0.04) | 16 | 0.16 (0.04) | **0.013** | 21 | 0.11 (0.03) | 14 | 0.11 (0.03) | 0.972 | **0.035** | **<0.001†** |
|  | *FADS2* | rs174583 | *C/T* | 19 | 0.13 (0.04) | 27 | 0.15 (0.04) | **0.033** | 13 | 0.12 (0.03) | 25 | 0.11 (0.04) | 0.580 | 0.498 | **0.001†** |
|  | *FADS2* | rs99780 | *C/T* | 15 | 0.13 (0.04) | 26 | 0.15 (0.04) | 0.060 | 12 | 0.12 (0.03) | 22 | 0.11 (0.04) | 0.584 | 0.576 | **0.001†** |
|  | *FADS2* | rs174602 | *T/C* | 18 | 0.13 (0.04) | 12 | 0.14 (0.034) | 0.539 | 23 | 0.11 (0.04) | 13 | 0.11 (0.04) | 0.684 | 0.133 | 0.142 |
| **C20:5n-3 (EPA)** | | | | | | | | | | | | | | | |
|  | *FADS2* | rs1535 | *A/G* | 19 | 0.33 (0.15) | 25 | 0.33 (0.14) | 0.912 | 12 | 0.26 (0.10) | 23 | 0.25 (0.19) | 0.975 | 0.132 | 0.136 |
|  | *FADS2* | rs174575 | *C/G* | 24 | 0.30 (0.14) | 16 | 0.37 (0.13) | 0.129 | 20 | 0.22 (0.10) | 14 | 0.28 (0.22) | 0.293 | 0.061 | 0.220 |
|  | *FADS2* | rs174583 | *C/T* | 19 | 0.33 (0.15) | 27 | 0.33 (0.13) | 0.974 | 12 | 0.26 (0.10) | 25 | 0.25 (0.19) | 0.937 | 0.132 | 0.074 |
|  | *FADS2* | rs99780 | *C/T* | 15 | 0.29 (0.14) | 26 | 0.35 (0.14) | 0.212 | 11 | 0.25 (0.10) | 22 | 0.27 (0.19) | 0.748 | 0.388 | 0.108 |
|  | *FADS2* | rs174602 | *T/C* | 19 | 0.31 (0.14) | 12 | 0.32 (0.10) | 0.780 | 23 | 0.23 (0.09) | 14 | 0.27 (0.13) | 0.347 | **0.040** | 0.241 |
| **C22:5n-3 (DPAn3)** | | |  |  |  |  |  |  |  |  |  |  |  |  |  |
|  | *FADS2* | rs1535 | *A/G* | 20 | 0.41 (0.10) | 25 | 0.42 (0.06) | 0.730 | 13 | 0.42 (0.11) | 23 | 0.37 (0.10) | 0.190 | 0.887 | **0.034** |
|  | *FADS2* | rs174575 | *C/G* | 24 | 0.40 (0.09) | 16 | 0.44 (0.05) | 0.056 | 21 | 0.39 (0.11) | 14 | 0.37 (0.10) | 0.632 | 0.872 | **0.033** |
|  | *FADS2* | rs174583 | *C/T* | 19 | 0.40 (0.10) | 27 | 0.42 (0.06) | 0.388 | 13 | 0.42 (0.11) | 25 | 0.37 (0.10) | 0.158 | 0.674 | **0.018** |
|  | *FADS2* | rs99780 | *C/T* | 15 | 0.38 (0.07) | 26 | 0.44 (0.08) | **0.017** | 12 | 0.42 (0.12) | 22 | 0.39 (0.10) | 0.429 | 0.297 | **0.043** |
|  | *FADS2* | rs174602 | *T/C* | 19 | 0.40 (0.10) | 13 | 0.43 (0.08) | 0.386 | 23 | 0.38 (0.10) | 14 | 0.38 (0.10) | 0.805 | 0.641 | 0.158 |
| **C22:6n-3 (DHA)** | | | | | | | | | | | | | | | |
|  | *FADS2* | rs1535 | *A/G* | 20 | 4.48 (1.35) | 25 | 4.23 (1.04) | 0.496 | 13 | 4.56 (0.98) | 23 | 4.18 (0.93) | 0.255 | 0.841 | 0.869 |
|  | *FADS2* | rs174575 | *C/G* | 24 | 4.19 (1.22) | 16 | 4.50 (1.02) | 0.400 | 21 | 4.25 (0.92) | 14 | 4.44 (1.01) | 0.573 | 0.856 | 0.859 |
|  | *FADS2* | rs174583 | *C/T* | 19 | 4.31 (1.16) | 27 | 4.29 (1.06) | 0.955 | 13 | 4.56 (0.98) | 25 | 4.11 (0.93) | 0.169 | 0.528 | 0.511 |
|  | *FADS2* | rs99780 | *C/T* | 15 | 4.14 (0.95) | 26 | 4.33 (1.19) | 0.599 | 12 | 4.64 (0.98) | 22 | 4.26 (0.88) | 0.260 | 0.188 | 0.835 |
|  | *FADS2* | rs174602 | *T/C* | 19 | 4.32 (1.09) | 13 | 4.52 (1.22) | 0.635 | 23 | 4.10 (0.91) | 14 | 4.19 (1.02) | 0.794 | 0.480 | 0.450 |
| P-value derived from global ANOVA and significance level (p<0.004) was adjusted for multiple testing by Bonferroni correction. Data are means of FAs expressed as percentages of the total phospholipid profile (standard error). P-values <0.05 are highlighted in bold and significant associations that persisted after Bonferroni correction are additionally denoted by stars or daggers (p<0.004). *Indicates significant differences within each group of weight and † Indicates significant differences between groups of weight. Major allele: M; minor allele: m; LA: Linoleic Acid; GLA: γ-Linolenic Acid; DGLA: Dihomo-γ-Linolenic Acid; AA: Arachidonic Acid; AdA: Adrenic Acid; DPAn6: Docosapentaenoic acid n6; ALA: α-linolenic Acid; EPA: Eicosapentaenoic acid; DPAn3: Docosapentaenoic acid n3; DHA: Docosahexaenoic acid. | | | | | | | | | | | | | | | |

e) Fatty acids involved in *ELOVL* indexes

|  | ***Gene*** | **SNP *Major/minor allele*** | | **NORMAL-WEIGHT** | | | | | **OVERWEIGHT/OBESITY** | | | | | **P (*MM*)** | **P (*Mm+mm*)** |
| --- | --- | --- | --- | --- | --- | --- | --- | --- | --- | --- | --- | --- | --- | --- | --- |
|  |  |  |  | ***MM*** | | ***Mm+mm*** | | **P** | ***MM*** | | ***Mm+mm*** | | **P** |  |  |
|  |  |  |  | **N** | **Mean (SD)** | **N** | **Mean (SD)** |  | **N** | **Mean (SD)** | **N** | **Mean (SD)** |  |  |  |
| **Fatty acids involved in *ELOVL2* indexes** | | | | | | | | | | | | | | | |
| **C22:4n-6 (AdA)** | | | | | | | | | | | | | | | |
|  | *ELOVL2* | rs2236212 | *G/C* | 14 | 0.38 (0.06) | 29 | 0.41 (0.10) | 0.396 | 13 | 0.38 (0.11) | 22 | 0.42 (0.09) | 0.253 | 0.941 | 0.540 |
|  | *ELOVL2* | rs3798713 | *G/C* | 11 | 0.37 (0.07) | 31 | 0.40 (0.10) | 0.363 | 12 | 0.39 (0.11) | 25 | 0.42 (0.08) | 0.377 | 0.739 | 0.576 |
|  | *ELOVL2* | rs953413 | *A/G* | 10 | 0.43 (0.14) | 26 | 0.39 (0.06) | 0.225 | 7 | 0.46 (0.11) | 20 | 0.39 (0.09) | 0.139 | 0.714 | 0.957 |
| **C22:5n-6 (DPAn6)** | | |  |  |  |  |  |  |  |  |  |  |  |  |  |
|  | *ELOVL2* | rs2236212 | *G/C* | 14 | 0.49 (0.12) | 29 | 0.52 (0.16) | 0.538 | 13 | 0.49 (0.16) | 22 | 0.53 (0.16) | 0.456 | 0.957 | 0.832 |
|  | *ELOVL2* | rs3798713 | *G/C* | 11 | 0.48 (0.11) | 31 | 0.50 (0.14) | 0.652 | 12 | 0.49 (0.17) | 25 | 0.52 (0.15) | 0.595 | 0.851 | 0.618 |
|  | *ELOVL2* | rs953413 | *A/G* | 10 | 0.50 (0.16) | 26 | 0.51 (0.13) | 0.898 | 7 | 0.60 (0.13) | 20 | 0.49 (0.16) | 0.113 | 0.207 | 0.635 |
| **C20:5n-3 (EPA)** | | | | | | | | | | | | | | | |
|  | *ELOVL2* | rs2236212 | *G/C* | 14 | 0.31 (0.12) | 29 | 0.33 (0.15) | 0.776 | 13 | 0.27 (0.23) | 22 | 0.24 (0.11) | 0.612 | 0.571 | **0.032** |
|  | *ELOVL2* | rs3798713 | *G/C* | 11 | 0.32 (0.13) | 31 | 0.34 (0.15) | 0.765 | 12 | 0.28 (0.24) | 25 | 0.24 (0.11) | 0.490 | 0.626 | **0.011** |
|  | *ELOVL2* | rs953413 | *A/G* | 10 | 0.30 (0.16) | 26 | 0.32 (0.13) | 0.611 | 7 | 0.22 (0.11) | 20 | 0.27 (0.19) | 0.513 | 0.258 | 0.243 |
| **C22:5n-3 (DPAn3)** | | |  |  |  |  |  |  |  |  |  |  |  |  |  |
|  | *ELOVL2* | rs2236212 | *G/C* | 14 | 0.43 (0.10) | 29 | 0.41 (0.07) | 0.424 | 13 | 0.34 (0.06) | 22 | 0.41 (0.12) | **0.047** | **0.011** | 0.787 |
|  | *ELOVL2* | rs3798713 | *G/C* | 11 | 0.43 (0.11) | 31 | 0.41 (0.07) | 0.465 | 12 | 0.35 (0.06) | 25 | 0.41 (0.12) | 0.099 | **0.033** | 0.992 |
|  | *ELOVL2* | rs953413 | *A/G* | 10 | 0.39 (0.06) | 26 | 0.41 (0.09) | 0.475 | 7 | 0.38 (0.10) | 20 | 0.39 (0.11) | 0.758 | 0.711 | 0.466 |
| **C22:6n-3 (DHA)** | | | | | | | | | | | | | | | |
|  | *ELOVL2* | rs2236212 | *G/C* | 14 | 4.64 (1.32) | 29 | 4.10 (0.98) | 0.139 | 13 | 4.30 (0.99) | 22 | 4.28 (0.97) | 0.942 | 0.466 | 0.514 |
|  | *ELOVL2* | rs3798713 | *G/C* | 11 | 4.60 (1.49) | 31 | 4.10 (0.91) | 0.200 | 12 | 4.41 (0.94) | 25 | 4.24 (0.96) | 0.617 | 0.728 | 0.565 |
|  | *ELOVL2* | rs953413 | *A/G* | 10 | 3.82 (0.83) | 26 | 4.31 (1.22) | 0.250 | 7 | 3.85 (0.73) | 20 | 4.61 (0.92) | 0.059 | 0.929 | 0.357 |
| **Fatty acids involved in *ELOVL5* indexes** | | | | | | | | | | | | | | | |
| **C18:2n6 (LA)** | |  |  |  |  |  |  |  |  |  |  |  |  |  |  |
|  | *ELOVL5* | rs2397142 | *C/G* | 24 | 23.49 (2.14) | 22 | 23.65 (2.13) | 0.806 | 19 | 23.41 (2.47) | 17 | 23.57 (2.20) | 0.839 | 0.906 | 0.911 |
|  | *ELOVL5* | rs9395855 | *T/G* | 7 | 23.31 (2.70) | 25 | 23.76 (2.36) | 0.668 | 8 | 23.54 (2.04) | 21 | 23.02 (2.38) | 0.587 | 0.854 | 0.294 |
| **C18:3n6 (GLA)** | | | | | | | | | | | | | | | |
|  | *ELOVL5* | rs2397142 | *C/G* | 22 | 0.05 (0.02) | 22 | 0.06 (0.02) | 0.684 | 19 | 0.05 (0.02) | 16 | 0.05 (0.02) | 0.787 | 0.787 | 0.571 |
|  | *ELOVL5* | rs9395855 | *T/G* | 7 | 0.06 (0.03) | 23 | 0.06 (0.02) | 0.990 | 8 | 0.05 (0.02) | 20 | 0.05 (0.02) | 0.653 | 0.557 | 0.496 |
| **C20:3n-6 (DGLA)** | | |  |  |  |  |  |  |  |  |  |  |  |  |  |
|  | *ELOVL5* | rs2397142 | *C/G* | 24 | 3.96 (0.86) | 22 | 3.76 (0.89) | 0.450 | 19 | 3.78 (1.17) | 17 | 3.95 (0.66) | 0.554 | 0.554 | 0.469 |
|  | *ELOVL5* | rs9395855 | *T/G* | 7 | 4.38 (0.87) | 25 | 3.82 (0.77) | 0.107 | 8 | 4.05 (0.88) | 21 | 3.86 (1.08) | 0.663 | 0.479 | 0.881 |
| **C20:4n-6 (AA)** | | | | | | | | | | | | | | | |
|  | *ELOVL5* | rs2397142 | *C/G* | 24 | 9.87 (1.58) | 22 | 9.16 (1.51) | 0.128 | 19 | 10.46 (1.16) | 17 | 9.90 (1.29) | 0.176 | 0.176 | 0.112 |
|  | *ELOVL5* | rs9395855 | *T/G* | 7 | 9.10 (1.86) | 25 | 9.93 (1.52) | 0.232 | 8 | 9.63 (1.26) | 21 | 10.39 (1.21) | 0.145 | 0.528 | 0.273 |
| **C22:4n-6 (AdA)** | | | | | | | | | | | | | | | |
|  | *ELOVL5* | rs2397142 | *C/G* | 24 | 0.40 (0.07) | 22 | 0.38 (0.11) | 0.402 | 18 | 0.40 (0.08) | 17 | 0.41 (0.10) | 0.977 | 0.977 | 0.420 |
|  | *ELOVL5* | rs9395855 | *T/G* | 7 | 0.39 (0.07) | 25 | 0.41 (0.10) | 0.608 | 8 | 0.43 (0.13) | 20 | 0.40 (0.08) | 0.452 | 0.524 | 0.566 |
| **C18:3n-3 (ALA)** | | | | | | | | | | | | | | | |
|  | *ELOVL5* | rs2397142 | *C/G* | 23 | 0.13 (0.04) | 20 | 0.15 (0.04) | 0.168 | 19 | 0.11 (0.03) | 16 | 0.11 (0.04) | 0.605 | **0.022** | **0.016** |
|  | *ELOVL5* | rs9395855 | *T/G* | 6 | 0.16 (0.05) | 25 | 0.14 (0.04) | 0.405 | 8 | 0.10 (0.02) | 20 | 0.11 (0.04) | 0.519 | **0.010** | **0.047** |
| **C20:5n-3 (EPA)** | | | | | | | | | | | | | | | |
|  | *ELOVL5* | rs2397142 | *C/G* | 24 | 0.33 (0.14) | 22 | 0.34 (0.14) | 0.855 | 18 | 0.21 (0.09) | 17 | 0.29 (0.22) | 0.168 | **0.004†** | 0.411 |
|  | *ELOVL5* | rs9395855 | *T/G* | 7 | 0.30 (0.15) | 25 | 0.31 (0.13) | 0.916 | 8 | 0.33 (0.29) | 20 | 0.25 (0.11) | 0.280 | 0.844 | 0.089 |
| P-value derived from global ANOVA and significance level (p<0.004) was adjusted for multiple testing by Bonferroni correction. Data are means of FAs expressed as percentages of the total phospholipid profile (standard error). P-values <0.05 are highlighted in bold and significant associations that persisted after Bonferroni correction are additionally denoted by stars or daggers (p<0.004). *Indicates significant differences within each group of weight and † Indicates significant differences between groups of weight. Major allele: M; minor allele: m; LA: Linoleic Acid; GLA: γ-Linolenic Acid; DGLA: Dihomo-γ-Linolenic Acid; AA: Arachidonic Acid; AdA: Adrenic Acid; DPAn6: Docosapentaenoic acid n6; ALA: α-linolenic Acid; EPA: Eicosapentaenoic acid; DPAn3: Docosapentaenoic acid n3; DHA: Docosahexaenoic acid. | | | | | | | | | | | | | | | |
