# Supplementary material for: Association of maternal weight with FADS and ELOVL genetic variants and fatty acid levels- The PREOBE follow-up
Source: PLoS One. 2017 Jun 9;12(6):e0179135. doi: 10.1371/journal.pone.0179135 (PMC5466308; doi:10.1371/journal.pone.0179135)
Supplement: S5 Table — (DOCX) [file pone.0179135.s006.docx]

S5 Table. Maternal EPA:AA and DHA:AA ratios in plasma according to their genotypes.

|  | ***Gene*** | **SNP *Major/minor allele*** | | **NORMAL-WEIGHT** | | | | | | **OVERWEIGHT/OBESITY** | | | | | **P (*MM*)** | **P (*Mm+mm*)** |
| --- | --- | --- | --- | --- | --- | --- | --- | --- | --- | --- | --- | --- | --- | --- | --- | --- |
|  |  |  |  | ***MM*** | | ***Mm+mm*** | | **P** | ***MM*** | | | ***Mm+mm*** | | **P** |  |  |
|  |  |  |  | **N** | **Mean (SD)** | **N** | **Mean (SD)** |  | **N** | | **Mean (SD)** | **N** | **Mean (SD)** |  |  |  |
| **Indexes** | | | | | | | | | | | | | | | | |
| **EPA:AA** | | | | | | | | | | | | | | | | |
|  | *FADS1* | rs174537 | *G/T* | 15 | 0.03 (0.02) | 23 | 0.04 (0.02) | 0.218 | 12 | | 0.02 (0.01) | 21 | 0.03 (0.02) | 0.690 | 0.231 | **0.040** |
|  | *FADS1* | rs174545 | *C/G* | 22 | 0.03 (0.02) | 27 | 0.04 (0.02) | 0.221 | 13 | | 0.02 (0.01) | 22 | 0.03 (0.02) | 0.663 | 0.108 | **0.031** |
|  | *FADS1* | rs174546 | *C/T* | 22 | 0.03 (0.02) | 27 | 0.04 (0.02) | 0.221 | 13 | | 0.02 (0.01) | 25 | 0.03 (0.02) | 0.728 | 0.108 | **0.016** |
|  | *FADS1* | rs174548 | *C/G* | 24 | 0.03 (0.02) | 25 | 0.04 (0.02) | 0.173 | 13 | | 0.02 (0.01) | 25 | 0.03 (0.02) | 0.899 | 0.135 | **0.013** |
|  | *FADS1* | rs174553 | *A/G* | 22 | 0.03 (0.02) | 27 | 0.04 (0.02) | 0.221 | 13 | | 0.02 (0.01) | 25 | 0.03 (0.02) | 0.728 | 0.108 | **0.016** |
|  | *FADS1* | rs174561 | *T/C* | 15 | 0.03 (0.02) | 14 | 0.03 (0.01) | 0.991 | 20 | | 0.03 (0.01) | 17 | 0.02 (0.01) | 0.375 | 0.100 | **0.010** |
|  | *FADS1* | rs174547 | *T/C* | 15 | 0.03 (0.02) | 23 | 0.04 (0.01) | 0.561 | 20 | | 0.02 (0.01) | 17 | 0.03 (0.02) | 0.634 | 0.143 | 0.097 |
|  | *FADS2* | rs1535 | *A/G* | 20 | 0.03 (0.02) | 25 | 0.04 (0.02) | 0.434 | 13 | | 0.02 (0.01) | 23 | 0.03 (0.02) | 0.703 | 0.092 | **0.045** |
|  | *FADS2* | rs174575 | *C/G* | 24 | 0.03 (0.02) | 16 | 0.04 (0.02) | **0.029** | 21 | | 0.02 (0.01) | 14 | 0.03 (0.02) | 0.165 | **0.029** | 0.088 |
|  | *FADS2* | rs174583 | *C/T* | 19 | 0.03 (0.02) | 27 | 0.04 (0.02) | 0.308 | 13 | | 0.02 (0.01) | 25 | 0.03 (0.02) | 0.728 | 0.092 | **0.016** |
|  | *FADS2* | rs99780 | *C/T* | 15 | 0.03 (0.02) | 26 | 0.04 (0.02) | 0.087 | 12 | | 0.02 (0.01) | 22 | 0.03 (0.02) | 0.529 | 0.300 | **0.033** |
|  | *FADS2* | rs174602 | *T/C* | 19 | 0.03 (0.02) | 13 | 0.03 (0.01) | 0.793 | 23 | | 0.02 (0.01) | 14 | 0.03 (0.01) | 0.206 | **0.014** | 0.242 |
|  | *ELOVL2* | rs2236212 | *G/C* | 14 | 0.03 (0.01) | 29 | 0.04 (0.02) | 0.605 | 13 | | 0.03 (0.02) | 22 | 0.02 (0.01) | 0.427 | 0.517 | **0.011** |
|  | *ELOVL2* | rs3798713 | *G/C* | 11 | 0.03 (0.01) | 31 | 0.04 (0.02) | 0.638 | 12 | | 0.03 (0.02) | 25 | 0.02 (0.01) | 0.348 | 0.554 | **0.004†** |
|  | *ELOVL2* | rs953413 | *A/G* | 10 | 0.03 (0.01) | 26 | 0.03 (0.02) | 0.483 | 7 | | 0.02 (0.01) | 20 | 0.03 (0.02) | 0.455 | 0.129 | 0.137 |
|  | *ELOVL5* | rs2397142 | *C/G* | 24 | 0.04 (0.02) | 22 | 0.04 (0.02) | 0.564 | 19 | | 0.02 (0.01) | 17 | 0.03 (0.02) | 0.102 | **0.005** | 0.161 |
|  | *ELOVL5* | rs9395855 | *T/G* | 7 | 0.04 (0.02) | 25 | 0.03 (0.01) | 0.372 | 8 | | 0.03 (0.03) | 21 | 0.02 (0.01) | 0.203 | 0.768 | **0.047** |
| **DHA:AA** | |  |  |  |  |  |  |  |  | |  |  |  |  |  |  |
|  | *FADS1* | rs174537 | *G/T* | 15 | 0.41 (0.16) | 23 | 0.47 (0.12) | 0.241 | 12 | | 0.43 (0.10) | 21 | 0.43 (0.10) | 0.995 | 0.839 | 0.197 |
|  | *FADS1* | rs174545 | *C/G* | 22 | 0.45 (0.16) | 27 | 0.49 (0.13) | 0.287 | 13 | | 0.43 (0.09) | 22 | 0.43 (0.10) | 0.988 | 0.772 | 0.091 |
|  | *FADS1* | rs174546 | *C/T* | 22 | 0.45 (0.16) | 27 | 0.49 (0.13) | 0.287 | 13 | | 0.43 (0.09) | 25 | 0.42 (0.10) | 0.753 | 0.772 | **0.039** |
|  | *FADS1* | rs174548 | *C/G* | 24 | 0.43 (0.13) | 25 | 0.51 (0.14) | **0.032** | 13 | | 0.45 (0.09) | 25 | 0.41 (0.10) | 0.276 | 0.586 | **0.005** |
|  | *FADS1* | rs174553 | *A/G* | 22 | 0.45 (0.16) | 27 | 0.49 (0.13) | 0.287 | 13 | | 0.43 (0.09) | 25 | 0.42 (0.10) | 0.753 | 0.772 | **0.039** |
|  | *FADS1* | rs174561 | *T/C* | 15 | 0.44 (0.14) | 14 | 0.51 (0.14) | 0.242 | 20 | | 0.40 (0.12) | 17 | 0.41 (0.08) | 0.809 | 0.350 | **0.024** |
|  | *FADS1* | rs174547 | *T/C* | 15 | 0.42 (0.16) | 23 | 0.47 (0.12) | 0.293 | 12 | | 0.43 (0.10) | 20 | 0.43 (0.10) | 0.835 | 0.871 | 0.330 |
|  | *FADS2* | rs1535 | *A/G* | 20 | 0.45 (0.16) | 25 | 0.47 (0.12) | 0.505 | 13 | | 0.43 (0.09) | 23 | 0.43 (0.10) | 0.916 | 0.764 | 0.148 |
|  | *FADS2* | rs174575 | *C/G* | 24 | 0.43 (0.14) | 16 | 0.51 (0.14) | 0.091 | 21 | | 0.41 (0.09) | 14 | 0.45 (0.11) | 0.229 | 0.636 | 0.239 |
|  | *FADS2* | rs174583 | *C/T* | 19 | 0.43 (0.15) | 27 | 0.49 (0.13) | 0.157 | 13 | | 0.43 (0.09) | 25 | 0.42 (0.10) | 0.753 | 0.971 | **0.039** |
|  | *FADS2* | rs99780 | *C/T* | 15 | 0.41 (0.14) | 26 | 0.48 (0.13) | 0.118 | 12 | | 0.44 (0.09) | 22 | 0.43 (0.10) | 0.796 | 0.521 | 0.163 |
|  | *FADS2* | rs174602 | *T/C* | 19 | 0.46 (0.16) | 13 | 0.47 (0.13) | 0.760 | 23 | | 0.40 (0.09) | 14 | 0.43 (0.12) | 0.387 | 0.117 | 0.322 |
|  | *ELOVL2* | rs2236212 | *G/C* | 14 | 0.48 (0.13) | 29 | 0.44 (0.14) | 0.305 | 13 | | 0.44 (0.12) | 22 | 0.41 (0.09) | 0.333 | 0.415 | 0.424 |
|  | *ELOVL2* | rs3798713 | *G/C* | 11 | 0.48 (0.14) | 31 | 0.44 (0.13) | 0.387 | 12 | | 0.46 (0.11) | 25 | 0.41 (0.09) | 0.184 | 0.643 | 0.384 |
|  | *ELOVL2* | rs953413 | *A/G* | 10 | 0.40 (0.08) | 26 | 0.46 (0.16) | 0.308 | 7 | | 0.38 (0.06) | 20 | 0.46 (0.09) | 0.055 | 0.588 | 0.921 |
|  | *ELOVL5* | rs2397142 | *C/G* | 24 | 0.44 (0.13) | 22 | 0.49 (0.15) | 0.287 | 19 | | 0.42 (0.09) | 17 | 0.44 (0.10) | 0.546 | 0.449 | 0.215 |
|  | *ELOVL5* | rs9395855 | *T/G* | 7 | 0.49 (0.20) | 25 | 0.43 (0.14) | 0.402 | 8 | | 0.44 (0.12) | 21 | 0.44 (0.09) | 0.864 | 0.599 | 0.898 |
| P-value derived from global ANOVA and significance level was adjusted for multiple testing by Bonferroni correction. Data are means of FAs expressed as percentages of the total phospholipid profile (standard error). P-values <0.05 are highlighted in bold and significant associations that persisted after Bonferroni correction are additionally denoted by stars or daggers (p<0.004). *Indicates significant differences within each group of weight and † Indicates significant differences between groups of weight. Major allele: M; minor allele: m; AA: Arachidonic acid; EPA: Eicosapentaenoic acid; DHA: Docosahexaenoic acid. | | | | | | | | | | | | | | | | |
